# Effectiveness and Safety of Shengmai San for Viral Myocarditis: A Systematic Review and Meta-Analysis of Randomized Controlled Trials

**DOI:** 10.1155/2024/2127018

**Published:** 2024-06-20

**Authors:** Bing-rui Zhang, Xue-han Liu, Yu-tong Ling, Chun-li Lu, Xin-yan Jin, Yi-ming Wei, Yi-qing Cai, Nicola Robinson, Jian-ping Liu

**Affiliations:** ^1^ Centre for Evidence-Based Chinese Medicine Beijing University of Chinese Medicine, Beijing 100029, China; ^2^ Dongzhimen Hospital Beijing University of Chinese Medicine, Beijing 100700, China; ^3^ Dongfang Hospital Beijing University of Chinese Medicine, Beijing 100078, China; ^4^ Guangdong Provincial Research Center of Integration of Traditional Chinese Medicine and Western Medicine in Metabolic Diseases (Institute of Chinese Medicine) Guangdong Pharmaceutical University, Guangzhou 510006, China; ^5^ Intensive Care Unit Beijing Hospital of Traditional Chinese Medicine, Beijing 100010, China; ^6^ Institute of Health and Social Care London South Bank University, London, UK

**Keywords:** meta-analysis, shengmai san, systematic review, traditional chinese medicine, viral myocarditis

## Abstract

**Background:** Shengmai San (SMS) is a traditional Chinese medicine formula used for supplementing *Qi* and *Yin* and can mitigate symptoms related to malignant arrhythmia and heart failure. This systematic review aimed at exploring the effectiveness and safety of SMS for viral myocarditis (VMC).

**Methods:** Eight databases from their inception to June 2023 were searched to identified randomized controlled trials (RCTs) focusing on SMS for VMC. The Cochrane Risk of Bias Tool was used to assess methodological quality. Mean difference (MD), standardized mean difference (SMD), and risk ratio (RR) with 95% confidence interval (CI) were calculated and input into the meta-analysis using RevMan 5.4.

**Results:** Forty-four RCTs were included involving 4298 participants. The interventions included 29 types of modified SMS decoctions and 15 patent medicines. Overall study quality was low. Compared with western medicine (WM), SMS was associated with higher recovery rate from palpitations (RR 2.3, 95% CI 1.59, 3.33, 2 RCTs, *n* = 89), chest pain (RR 1.57, 95% CI [1.17, 2.09], 2 RCTs, *n* = 89), and lower cTnI (MD −0.82 ng/ml, 95% CI −0.98, −0.66, 1 RCT, *n* = 60). SMS plus WM was more effective than WM in palpitation recovery rate (RR 1.52, 95% CI 1.21, 1.92, 3 RCTs, *n* = 136), dyspnea recovery rate (RR 1.47, 95% CI 1.12, 1.94, 3 RCT, *n* = 267), ECG (RR 1.43, 95% CI 1.32, 1.55, 20 RCT, *n* = 2035), CK-MB (MD −6.36, 95% CI −8.43, −4.28, 8 RCT, *n* = 946), and cTnI (MD −0.06, 95% CI −0.06, −0.05, 3 RCT, *n* = 307). No serious adverse events were reported using SMS alone or in combination with WM.

**Conclusion:** SMS used alone or combined with WM may have potential effectiveness on symptom alleviation, ECG recovery rate, myocardial injury markers, and cardiac function, but the effectiveness is uncertain due to the low quality and absence of placebo-controlled trials. The exact efficacy of SMS for VMC needs to be confirmed by high-quality double-blind RCTs in the future.

## 1. Introduction

Viral myocarditis (VMC) is an inflammatory disease of myocardium caused by viral infections and characterized by inflammatory infiltration and myocardial damage [1]. Almost all human virus infections involve myocardium. More than 30 kinds of virus have been found harmful in myocarditis, with Coxsackie virus being the most common [2]. Estimates suggest that the incidence of VMC is increasing, 0.022% (1990–2013) and 0.04% (1998–2017), with a 4.16% all-cause mortality in all English National Health Service hospitals in 2017 [3, 4]. Myocarditis is the major cause of shock and dilated cardiomyopathy in young adults [5]. About 30% of hospitalized patients with COVID-19 had myocardial involvement [6], and the risk and 1-year burden of cardiovascular disease in survivors of acute COVID-19 were substantial [7]. The main mechanisms for the development of VMC are viral injury and immune response. VMC has the presentation varying from mild symptoms of chest pain, palpitation, dyspnea, and transient electrocardiogram (ECG) changes to life threatening incidents of cardiogenic shock and severe ventricular arrhythmia, often accompanied with upper respiratory tract infections or diarrhea 3 weeks before the onset [1]. Conventional treatments target preventing malignant arrhythmia and heart failure, but there are no targeted treatments for symptoms of palpitation, dyspnea, and chest pain. The Brazilian Society of Cardiology Guideline on Myocarditis 2022 [8] suggests that most patients with VMC who have spontaneous regression of clinical symptoms do not require therapeutic intervention. However, symptoms of palpitation, dyspnea, and chest pain can remain for a long time and impact quality of life.

Shengmai San (SMS) is a classical formula which first appeared in the Medical Enlightenment written by Zhang Yuansu in Jin Dynasty (about 1000 years ago) [9]. It consists of Renshen (*Panax ginseng C.A.Mey.*) (people today also use similar herbs Dangshen (*Codonopsis pilosula Nannf.*) or Taizishen (*Pseudostellaria heterophylla* (*Miq.*) *Pax*)), Maidong (*Ophiopogon japonicus* (*Thunb.*) *Ker Gawl.*), and Wuweizi (*Schisandra chinensis* (*Turcz.*) *Baill.*). SMS is used for *Deficiency of Qi and Yin*, one of the traditional Chinese medicine (TCM) syndrome types, manifesting with symptoms of palpitation, fatigue, chest tightness, shortness of breath, insomnia, being easily frightened, light red tongue, and weak pulse; this fits with symptoms of VMC. SMS has a long history of treating cardiac disease, with its effectiveness widely reported in clinical practice. A meta-analysis previously demonstrated that SMS may relieve symptoms and improve ECGs in patients with angina pectoris [10]. A systematic review [11] of randomized controlled trials (RCTs) of SMS for VMC published in Chinese included trials limited to decoctions and used “total effective rate” as the outcome measure, but this outcome was vaguely defined and inconsistent between trials. It lacked symptom-related outcomes, so it could not clearly explain the effectiveness of SMS. Therefore, we conducted a systematic review and meta-analysis including both decoctions and patent medicines based on SMS.

We evaluated from symptom disappearance rate (palpitation, dyspnea, and chest pain) to objective examinations (ECG, myocardial injury markers, and cardiac function) in order to explore the effectiveness and safety of SMS for VMC and evaluate the risk of bias of included studies to provide methodological recommendations for future research.

## 2. Methods

### 2.1. Registration

The protocol of this review was registered via PROSPERO (CRD42022382055) on the 17th of December 2022 (Available from:http://www.crd.york.ac.uk/PROSPERO/). The content of this review followed Preferred Reporting Items for Systematic Reviews and Meta-Analyses (PRISMA 2020) (details in Table S1).

### 2.2. Eligibility Criteria

#### 2.2.1. Type of Studies

RCTs were included in the systematic review, comparing SMS alone or combining SMS with conventional therapy and using conventional therapy, placebo, or no intervention as control.

#### 2.2.2. Type of Participants

Patients with clinically suspected or definite VMC were included, basing on current comprehensive criteria [1, 12–14] or as defined by the International Classification of Diseases (ICD-11): The clinically suspected cases of VMC were based on ≥ 1 clinical presentation (palpitation, chest pain, dyspnea, fatigue, etc.) and ≥ 1 diagnostic criteria using different categories (ECG changes, raised level of myocardial injury markers, and functional and structural abnormalities in cardiac imaging), while asymptomatic patients had to meet ≥ 2 diagnostic criteria, in the absence of other disease which could explain the syndrome. The definitive diagnosis of VMC was based on endomyocardial biopsy. Patients at any phase of the disease were included. There were no restrictions on age, gender, or ethnicity.

#### 2.2.3. Type of Intervention

Interventions included SMS decoctions or patent medicine (including Renshen (*Panax ginseng C.A.Mey.*), Dangshen (*Codonopsis pilosula Nannf.*), or Taizishen (*Pseudostellaria heterophylla* (*Miq.*) *Pax*); Maidong (*Ophiopogon japonicus* (*Thunb.*) *Ker Gawl.*); and Wuweizi (*Schisandra chinensis* (*Turcz.*) *Baill.*)), singly or combination with conventional therapy (e.g., clinical monitoring, antiviral therapy, arrhythmia-correcting therapy, and cardioprotective therapy, except for other TCM treatments). Additions and subtractions according to TCM syndrome differentiation were permissible, with no limitation on the quantity of additional herbs. Authors had to report decoctions or patent medicine that were based on SMS with dosages of each herb. Comparisons included conventional treatments (except other TCM), placebo, or no intervention.

#### 2.2.4. Type of Outcomes

Palpitations, dyspnea, and chest pain are the most common symptoms and are diagnostic criteria of VMC [6], so we use their disappearance rate as the primary outcome. Some high-quality clinical trials have used “symptom disappearance rate” as the primary outcome [15], which is commonly used in systematic review and meta-analysis [16].VMC can lead to complications such as heart failure, severe arrhythmias, cardiac shock, and pericardial effusion [1]. And 26% of patients with acute myocarditis present with complications such as left ventricular systolic dysfunction, sustained ventricular arrhythmias, and low cardiac output syndrome [17], so we use “incidence of complications” as the primary outcome. We use cardiac biomarkers including isoenzyme of creatine kinase MB (CK-MB), cardiac troponin I (cTnI), and N-terminal pro-B-type natriuretic peptide (NT-proBNP) to confirm the presence of increased myocardial wall stress and evidence of myonecrosis [18]. We use left ventricular ejection fraction (LVEF) and early to late diastolic transmitral flow velocity (E/A) to evaluate cardiac function. LVEF is an indicator for systolic function, and E/A is to assess ventricular diastolic function [19].

Primary outcomes included (1) disappearance rate of symptoms of palpitations, dyspnea, and chest pain and (2) incidence of complications (e.g., heart failure, severe arrhythmias (ventricular tachycardia, sinus arrest), cardiac shock, and pericardial effusion).

Secondary outcomes included (1) ECG recovery rate, (2) myocardial injury marker (e.g., CK-MB, cTnI, and NT-proBNP), (3) cardiac function (e.g., LVEF, E/A), and (4) the score of general symptoms [20] (e.g., TCM Syndrome Score).

Safety outcome included adverse events.

### 2.3. Search Strategy

A total of five Chinese databases were searched (China National Knowledge Infrastructure (CNKI), Wanfang Database, Chinese Scientific Journal Database (VIP), SinoMed, and Yiigle Database) and three English databases (PubMed, EMBASE, and The Cochrane Library) from their inception to June 25, 2023, for publications of journal articles, conference papers, and academic dissertations written in Chinese or English. Different searching strategies were applied for different databases (Table S2). We also hand-searched the references of relevant studies for additional eligible RCTs. Multiple publications referring to the same study were identified, but the most comprehensive one was included.

### 2.4. Study Selection and Data Extraction

Two authors (BRZ and YTL) screened titles and abstracts using NoteExpress 3.6.0 software to identify potentially eligible studies and downloaded full texts to judge eligible studies. Two authors (BRZ and YTL) extracted data independently from the included studies according to a predesigned data sheet (including publication years, fundings, inclusion/exclusion criteria, diagnostic criteria, characteristics of participants, details of intervention/control, and outcomes). Any differences were resolved by consensus or consulting a senior author (XHL).

### 2.5. Quality Assessment

We assessed the risk of bias independently and in duplicate using the Cochrane Risk of Bias 2.0 tool [21] in the following domains: randomization process, deviations from intended interventions, missing outcome data, measurement of the outcome, and selection of the reported result. We rated each domain as “low,” “some concerns,” or “high.” We determined the overall risk of bias for each trial based on the highest risk attributed to any one domain. For randomization process, only mentioning “randomized” meant “some concerns” risk of bias. Discrepancies in the judgement were resolved by consensus or consulting a senior author.

### 2.6. Data Analysis

We used meta-analysis with RevMan 5.4 software to analyze data. Mean difference (MD) and standard mean difference (SMD) with 95% confidence intervals (95% CI) were used for the analysis of continuous data. MD was used for outcomes reported by the same measurement. SMD was used when outcomes were reported by different measurements or scales. The relative risk (RR) with 95% CI was used for dichotomous outcomes. Literature that could not undergo meta-analysis was used to provide a descriptive summary.

The statistical heterogeneity between trials was quantified with I-square (*I*^2^). Due to differences in additional herbs, cooking individualization, and treating durations, we used a random effect model to combine the data. We conducted subgroup analysis based on the dosage form and quantity of additional herbs under each meta-analysis. When *I*^2^ > 50% with available data, we performed subgroup analysis based on (1) phases of VMC [13] (within 6 months, over 6 months), (2) duration of treatments (2 weeks, 1 month, or 3 months), (3) quantity of additional herbs according to TCM syndrome differentiation (none; ≤ 6 herbs; > 6 herbs), and (4) types of “Shen” (Renshen (*Panax ginseng C.A.Mey.*), Dangshen (*Codonopsis pilosula Nannf.*), and Taizishen (*Pseudostellaria heterophylla* (*Miq.*) *Pax*)).

When *I*^2^ > 50%, a sensitivity analysis was conducted based on study quality. A funnel plot test was generated to evaluate publication bias when more than 10 trials existed in a meta-analysis.

## 3. Results

### 3.1. Study Identification and Characteristics

**Figure 1:**
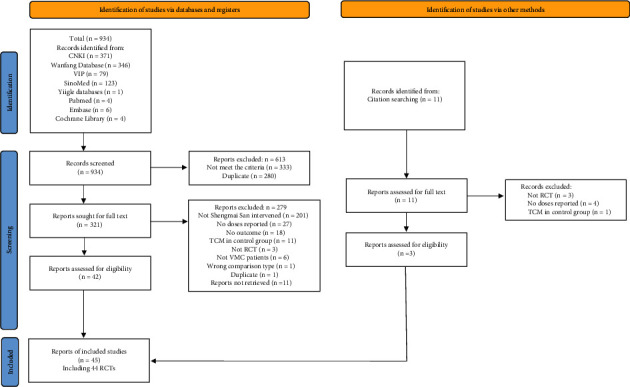
PRISMA flow diagram of the study selection process.

A total of 929 articles were identified, and an additional 11 were identified through hand-searches. The included 44 RCTs [22–65] contained 4298 patients. The screening process is shown in Figure 1. All studies were published in Chinese. The details of included 44 trials are presented in Table 1. All patients met clear diagnostic criteria for VMC, but types of virus were unknown in most studies. The sample sizes for individual trials ranged from 38 to 296. Seven trials [29, 30, 32, 39, 42, 50, 52] involved adolescents under 14, and 23 trials involved adults, and the other two trials [40, 60] involved both adolescents and adults, while 11 trials [22, 24, 31, 34, 37, 38, 49, 57, 59, 61] had no information on age. The male-to-female ratio was 2145/1855, except four trials [22, 24, 31, 57] failed to report gender. Five trials [27, 31, 33, 43, 53] included convalescent patients with VMC for more than 6 months.

The SMS interventions included 29 decoctions and 15 patent medicines. For SMS decoctions, 14 trials [25, 27, 43, 44, 46, 47, 53–57, 61, 64, 65] were based on SMS, and 13 trials [22, 26, 28, 29, 31, 32, 42, 45, 52, 59, 60, 62, 63] were based on SMS combined with other classical decoctions, and two trials [34, 39] used SMS alone with additions according to TCM syndrome differentiation. Patent medicines based on SMS included Shensong Yangxin Capsule (six trials [23, 24, 38, 40, 41, 58]), Yixinshu (five trials [36, 37, 48–50]), Huangqi Shengmaiyin (three trials [30, 33, 51]), and Lvfukang Capsule (one trial [35]). Compositions and dosages of SMS varied between trials, which are shown in Table S3. Treatments for control groups mainly included myocardial nutrients, antiviral drugs, and rhythmic normalization drugs. Only one trial [32] reported a follow-up visit at 3 months. Three different comparisons were considered: SMS versus WM, SMS plus WM versus WM, and SMS plus WM versus certain western medicine (cWM) plus WM.

### 3.2. Risk of Bias

**Figure 2:**
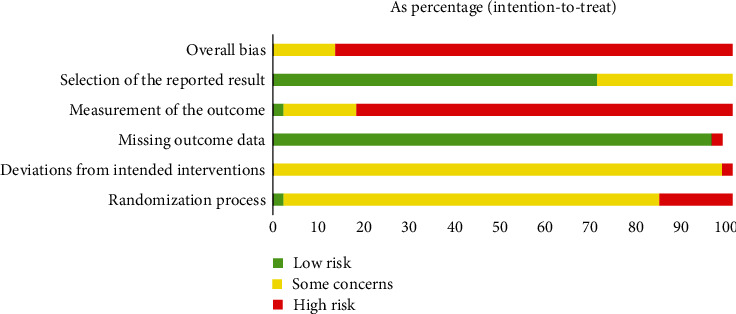
Summary of risk of bias.

The overall risk of bias of majority of included trials was “high” (Figure 2), while others were “some concerns.” Majority trials were marked “some concerns” in randomization process and deviations from intended interventions. No trial used blinding of participants and personnel. Seven trials [28, 29, 48–51, 64] reported generating random sequence by random number table or computer generated-list, and only one trial [65] reported the allocation concealment. Seven trials [25, 40, 43, 46, 47, 60, 62] had large difference between number of two groups, marked as “high” risk of bias in randomization process. For missing outcome data, only one trial [47] had dropouts over 5% without any analysis, marked as “high” risk of bias. For measurement of the outcome, none trial had blinding of outcome assessments and most trials measured symptoms, so most trials were marked “high” risk of bias, and only one trial [58] with objective measurement alone was marked “low” risk of bias. For selection of the reported result, most trials reported all preset outcomes or relatively complete outcomes, which were rated as “low” risk of bias. Thirteen trials did not report pre-set outcomes or reported over presetting, or the outcomes were brief and had no presetting, so their selection of the reported result was rated as “some concerns” risk of bias.

### 3.3. Primary Outcomes

Overall effects are shown in Table 2.

#### 3.3.1. Symptom Disappearance Rate

Compared with energy mixture, SMS was better in improving palpitation (RR 2.3, 95% CI 1.59–3.33; *N* = 2; *n* = 89) and chest pain (RR 1.57, 95% CI 1.17–2.09; *N* = 2; *n* = 89) disappearance [25, 26]. SMS plus WM was better than WM in improving disappearance of palpitation (RR 1.52, 95% CI 1.21–1.92; *N* = 3; *n* = 136) [32, 36, 46], dyspnea (RR 1.47, 95% CI 1.12–1.94; *N* = 3; *n* = 267) [32, 36, 46], and chest pain (RR 2.11, 95% CI 1.38–3.23, *N* = 1, *n* = 152) [46].

#### 3.3.2. Incidence of Complications

Only two trials [33, 58] reported complications. One trial [33] of SMS plus myocardial nutrient compared with myocardial nutrient reported no complication in both groups. One trial [58] reported 2 cases of sinus arrest in amiodarone hydrochloride plus usual care group but no complications in SMS plus usual care group.

### 3.4. Secondary Outcomes

#### 3.4.1. ECG Recovery Rate

SMS plus WM was better in improving ECG recovery rate compared with WM (RR 1.46, 95% CI 1.34–1.59; *N* = 20; *n* = 2035; Figure S1) [28, 30, 34, 36, 38, 40–42, 44–48, 50–52, 54–57] and cWM plus WM (RR 1.8, 95% CI 1.28–2.54; *N* = 6; *n* = 671) [59, 60, 62–65]. For patients with convalescent VMC, SMS plus myocardial nutrient had a higher ECG recovery rate compared with myocardial nutrient (RR 1.25, 95% CI 1.02–1.54; *N* = 2; *n* = 160) [33, 53]. But the result of meta-analysis did not show a significant benefit of SMS compared with WM (RR 1.33, 95% CI 0.85–2.09; *N* = 3; *n* = 173) [23, 25, 26].

#### 3.4.2. Myocardial Injury Marker

For a decrease in CK-MB, SMS plus WM showed a better effect compared with WM (MD −8.23 U/L, 95% CI −12.89 to −3.56; *N* = 9; *n* = 1060; *I*^2^ = 99%) [28, 29, 32, 36, 37, 40, 48, 49, 52], and the heterogeneity of decoctions subgroup reduced most after removing a low-quality trial [39] (MD −6.38 U/L, 95% CI −8.93 to −3.84; *N* = 4; *n* = 399; *I*^2^ = 88%) [28, 30, 32, 52]. Due to excessive heterogeneity, we conducted a subgroup analysis based on the quantity of additional herbs after excluding the low-quality trial (Figure S2). Subgroup analysis showed that more additional herbs may better reduce CK-MB, but large heterogeneity still existed probably due to methods and instrument differences of examinations. SMS and ribavirin had no difference in reducing CK-MB (MD 12.99 U/L, 95% CI −3.14–29.12; *N* = 1; *n* = 68) [24]. SMS plus WM was better in reducing CK-MB (MD −7.57 U/L, 95% CI −8.83 to −6.32; *N* = 3; *n* = 240) [64, 65, 69] than cWM plus WM.

For decrease of cTnI, SMS plus WM was better than WM (MD −0.06 ng/ml, 95% CI −0.06 to −0.05, *N* = 3; *n* = 307) [29, 32, 52] and cWM plus WM (MD −0.67 ng/ml, 95% CI −1.01 to −0.33; *N* = 1; *n* = 60) [59], and SMS was better than WM (MD −0.82 ng/ml, 95% CI −0.98 to −0.66; *N* = 1; *n* = 60) [22].

#### 3.4.3. Cardiac Function

SMS plus WM elevated LVEF more compared with cWM plus WM (MD 5.44%, 95% CI 1.56–9.33; *N* = 2; *n* = 180) [61, 64]. The results of RCTs indicated the benefit of SMS plus WM in LVEF elevation in both acute phase (MD 8.7%, 95% CI 4.69–12.71; *N* = 1; *n* = 62) [47] and convalescent phase (MD 5.85%, 95% CI 2.16-9.54; *N* = 1; *n* = 62) [43] compared with WM.

#### 3.4.4. The Score of General Symptoms

SMS plus WM had a better effect compared with WM (SMD −1.11, 95% CI −1.48 to −0.75; *N* = 3; *n* = 449; *I*^2^ = 69%) [28, 35, 45], and SMS plus usual care was better compared with creatine phosphate sodium plus usual care (MD −7.5, 95% CI −10.07 to −4.93; *N* = 2; *n* = 180; *I*^2^ = 87%) [64, 65] in the score of general symptoms, with a large heterogeneity probably related to evaluating differences. For convalescent VMC patients, SMS plus trimetazidine was better compared with trimetazidine (MD −6.3, 95% CI −7.05 to −5.55; *N* = 1; *n* = 60) [31].

### 3.5. Safety

A total of 14 trials [23, 24, 27, 28, 32, 35, 37, 39, 41, 52, 58, 63, 64] reported adverse effects, with no significant between-group difference of adverse incidence. No serious adverse event but tolerable symptoms of nausea, inappetence, dizziness, and headache happened in SMS groups. More serious adverse events including sinus arrest, liver function damage, and thyroid function abnormality happened in control groups. More details are shown in Table S4.

### 3.6. Publication Bias

We assessed the publication bias for ECG recovery rate comparing SMS plus WM with WM due to the limitation of the number of trials. We found the funnel plot roughly symmetrical, suggesting a small possibility of publication bias (Figure S3). According to all included studies, no English-language study may show potential publication bias.

## 4. Discussion

### 4.1. Summary of Findings

The main findings of this study indicated that SMS alone or combined with WM could improve the disappearance rate of palpitations, dyspnea, and chest pain compared with WM for patients with VMC. The secondary findings indicated that SMS combined with WM could improve ECG recovery rate, CK-MB, cTnI, LVEF, and general symptoms compared with WM (or adding cWM) for patients with VMC. SMS combined with WM showed a better effect on ECG recovery and CK-MB while SMS had no significant between-group difference with WM, which may be related to the synergy effect of SMS and WM. Therefore, SMS combined with WM may be a better choice for VMC patients with good safety.

Subgroup analysis suggested that decoctions have better effects on palpitation (SMS + WM vs WM), dyspnea (SMS + WM vs WM), and ECG recovery (SMS vs WM; SMS + WM vs WM of convalescence) compared with patent medicine. Some decoctions had additions according to TCM syndrome differentiation, and the decocting process allowed herbs to better synergize and assist, which may result in a better effect of decoctions. Subgroup analysis showed that additional herbs > 6 had better effects on CK-MB compared with additional herbs ≤ 6, which indicated that adequate additions according to TCM syndrome differentiation might produce a better effect.

We analyzed the use of frequency, property, and function of additional herbs (Figure S4). There were 71 additional drugs used in 44 RCTs, in which Danshen (*Salvia miltiorrhiza Bunge*, 31 RCTs) and Huangqi (*Eleutherococcus henryi Oliv.*, 28 RCTs) were the most frequently used. For their property, 44.2% are cold and 35.3% are warm, and their main functions are blood-circulating (38.5%), heat-clearing (36.9%), yin-nourishing (36.3%) and qi-tonifying (32.2%).

### 4.2. Compared with Previous Studies

A systematic review [66] included RCTs of herbal medicines for VMC, and it found one RCT where the SMS decoction plus supportive therapy significantly improved quality of life (SF-36) but had no significant difference on symptom improvement and abnormal ECG compared with supportive therapy, which had different outcomes with ours limiting by the number of studies included. A systematic review [11] of SMS for VMC included RCTs comparing SMS decoctions plus WM with WM, taking “total effective rate,” lactic dehydrogenase (LDH), CK, CK-MB, aspartate aminotransferase, ECG improvement, and adverse events as outcomes. Results of the included 26 RCTs showed that SMS plus WM had better effectiveness for VMC in “total effective rate,” LDH, CK, CK-MB, and ECG improvement compared with WM. However, the study [11] lacked symptom-related outcomes and the methodological quality of the systematic review was low and was less convincing.

### 4.3. Strengths and Limitations

For strengths, we were comprehensive in including studies wherever possible by considering all dosage forms (decoctions and patent medicines) and additions according to TCM syndrome differentiation. Regarding outcomes, we focused on symptom alleviation, which was the most concerning issue in clinical practice. We conducted meta-analysis separately under four different types of comparator, and we took convalescence patients for individual analysis. Patent medicine included capsule, pill, and oral liquid produced by pharmaceutical companies, and decoctions were made by pharmacy or patients. They are different in dosage form and drug content, so we conducted subgroup analysis based on dosage form under each meta-analysis. To explain the high heterogeneity, we conducted a subgroup analysis based on the quantity of additional herbs and conducted a sensitivity analysis.

For limitations, the included studies were all published in China, and none was placebo-controlled, with overall quality rated “low.” Clinical and methodological heterogeneity across trials in terms of SMS formulations used, control interventions, outcomes measured, follow-up periods, and so on reduced confidence in the pooled estimates. Complications and adverse events were not comprehensively reported, so it was difficult to explore the end-point outcomes and safety. We failed to explore the long-term effect of SMS for VMC because only one included trial reported a follow-up visit. We were unable to adequately assess the effect of SMS for acute or convalescent VMC because some trials failed to differentiate between the course of VMC. Due to lack of data, we had no outcomes for NT-proBNP or E/A. Age distribution of subjects varied between trials, and 11 trials did not report it, with absent data for subgroup analysis. Duration of treatment was mostly within 30 days, and four trials did not report duration, so we did not perform a subgroup analysis of treatment duration. There were no enough trials under single comparator for researchers to make a subgroup analysis based on types of “Shen.” Trials of patent medicine did not report dosage, so we could not analyze the additional herbs at dosage level.

### 4.4. Implications for Future Practice and Research

For future practice, SMS combined with WM may be a potential for VMC patients. Although the composition and dosage of the herbs included in each study were different, the included studies made prescriptions all based on SMS using the same TCM theory, so they can be analyzed in a meta-analysis. TCM believes that the mechanism of VMC is exogenous wind-heat-dampness and deficiency of *Qi-Yin*, and the treatment is clearing heat-toxin and tonifying *Qi-Yin*. VMC is caused from external wind-heat-dampness, and heat causes stasis-resolving, so blood-circulating and heat-clearing herbs may help. Heat decreases *Yin* of heart, so we use yin-nourishing herbs; yin deficiency results in yang deficiency, so qi-tonifying herbs may be used. Besides, clinicians should make personalized prescriptions according to TCM syndrome differentiation, for example, for patients with wind-heat, adding Jingyinhua (*Lonicera japonica Thunb.*) and Lianqiao (*Forsythia suspensa* (*Thunb.*) *Vahl*); for patients with blood-stasis, adding Honghua (*Carthamus tinctorius L.*) and Chishao (*Paeonia veitchii Lynch*); and for patients with qi deficiency, adding Huangqi (*Eleutherococcus henryi Oliv.*) and Longyanrou (*Dimocarpus longan Lour*.). The more comprehensive consideration of pathogenesis, the better the therapeutic effects may be.

In terms of pharmacological mechanism, animal experiment demonstrated that 20S-protopanaxatriol of *Panax ginseng C.A.Mey.* decreased virus titers and myocardial injury markers in mice [67], which had similar outcomes in our meta-analysis of myocardial injury marker. The interaction of complex components based on sodium taurocholate cotransporting polypeptide (NTCP) may be an important mechanism in SMS [68]. Network pharmacology predicted that [69] SMS intervenes in VMC by regulating cytokines, protein kinases, natural immune genes, intercellular adhesion molecules, and so on, and the mechanism includes TNF pathway, Toll-like receptor pathway, IL-17 pathway, and C-type lectin receptor pathway.

Future research should include reporting and recording complications such as dilated cardiomyopathy, pericardial effusion, and cardiogenic shock which determine the prognosis of VMC [6]. Multicenter, double-blind, placebo-controlled trials should be conducted to explore the effectiveness and safety of SMS for VMC with registered protocols, adverse event monitoring, and transparent reporting.

## 5. Conclusion

Low-certainty of preliminary evidence showed that SMS used alone or combined with WM may have potential effectiveness on symptom alleviation, ECG, myocardial injury markers, and cardiac function in patients with VMC. The exact efficacy of SMS for VMC needs to be confirmed by high-quality double-blind RCTs in the future.

## Figures and Tables

**Table 1:** Characteristics of included randomized clinical trials on SMS for VMC.

**Study ID**	**Sample size (S/W)**	**Age/year**	**Gender (M/F)**	**Comparisons**	**Treatment duration/day**	**Outcomes**
*SMS VS WM, 5 trials*						
Decoction, 3 RCTs						
Zhang and Chen [22]	30/30	NR	NR	Shengmai Xianxiong decoction bid vs GIK + Vit.C qd + CoQ Capsule tid	28	Total effectiveness; cTnI; T-lymphocyte subsets
Li [25]	30/25	S: 52.5 (29~67) W: 53.2 (29~67)	S: 13/17 W: 11/14	Qingxin Shengmai decoction bid vs AD + CoQ tid po	28	Disappearance rate of palpitation, chest tightness, chest pain, fatigue, and premature beat; total effectiveness; myocardial enzyme
Zhou [26]	38/20	S: 32.5 (15~44) W: 33.2 (14~47)	S: 22/16 W: 11/9	Shengmai Huangqiguizhi decoction tid vs AD + CoQ tid po	28	
Patent medicine, 2 RCTs						
Xu [23]	30/30	S: 18~40 W: 20~39	S: 16/14 W: 15/15	Rongxin Wan 4#+Shensong Yangxin Capsule 6# tid vs metoprolol succinate qd po	NR	TCM syndrome effectiveness; ECG; adverse events
Liu [24]	34/34	NR	NR	Shensong Yangxin Capsule 3# tid vs ribavirin + CoQ + Vit.C tid po	28	Total effectiveness; separate symptom score; ECG; adverse events

*SMS + WM VS WM, 26 trials*						
Decoction, 15 RCTs						
Yao, Gao, and Wei [28]	46/46	S: 34.65 ± 7.78 W: 32.97 ± 6.94	S: 27/19 W: 25/21	SMS plus Xiaochaihu decoction bid + CoA bid ivgtt.+ribavirin bid ivgtt.+Vit.C tid po vs CoA bid ivgtt.+ribavirin bid ivgtt.+Vit.C tid po	28	Total effectiveness; TCM Syndrome Score; myocardial enzyme; adverse events
Tan, Zhang, and Qiu [29]	40/40	S: 7.91 ± 2.82 W: 7.72 ± 2.75	S: 24/16 W: 22/18	Ermai Yangxin decoction tid + antibiotics + gamma globulin ivgtt.+Vit.E (large dose) + Vit.C + CoQ vs antibiotics + gamma globulin ivgtt.+Vit.E (large dose) + Vit.C + CoQ	7	Total effectiveness; myocardial enzyme
Li and Zhao [32]	55/56	S: 7.69 ± 3.07 W: 8.63 ± 3.15	S: 29/26 W: 26/30	Ermai Yangxin decoction tid + AD + antiviral drug and antibiotics + CoQ + Vit.E + Vit.C (large dose) vs AD + antiviral drug and antibiotics + CoQ + Vit.E + Vit.C (large dose)	30	Total effectiveness; myocardial enzyme; separate TCM syndrome recovery; adverse events
Sheng [34]	23/22	2~4: 8 5~10: 22 11~14: 15	24/21	SMS bid + AD + ribavirin qd ivgtt.+CoQ Capsule tid po vs AD + ribavirin qd ivgtt.+CoQ Capsule tid po	14	Total effectiveness
Zhou [39]	56/58	S: 5.4 ± 2.3 W: 5.7 ± 2.1	S: 31/25 W: 35/23	SMS 3–4/d + AD vs AD	15	Total effectiveness; myocardial enzyme; adverse events
Su and Zhu [44]	36/36	S: 34.67 ± 8.44 W: 35.78 ± 8.47	S: 19/17 W: 21/15	Qilian Shengmaiyin bid + GIK + CoQ tid po vs GIK + CoQ tid po	14	Total effectiveness; myocardial oxygen consumption; systolic blood pressure; heart rate
Zhu [42]	32/28	S: <5: 4, 6~10: 15, 11~14: 13. W: <5: 4, 6~10: 13, 11~14: 11	S: 19/13 W: 16/12	Shengmai Sanhuangjiedu decoction bid + GIK + penicillin (fosfomycin if allergic) + ribavirin vs GIK + penicillin (fosfomycin if allergic) + ribavirin	30	Total effectiveness
Tao and Wang [45]	93/93	S: 29.63 ± 11.21 W: 26.31 ± 10.12	S: 36/57 W: 38/55	SMS plus Zhigancao decoction tid + AD vs AD	28	Total effectiveness; effectiveness of symptoms and signs; TCM Syndrome Score; ECG; myocardial enzyme
Su and Zhou [46]	32/22	S: 20.5 ± 10.3 W: 21.3 ± 9.6	S: 20/12 W: 14/8	Shengmai Yuxin decoction bid + AD + Vit.C bid ivgtt.+CoQ tid po vs AD + Vit.C bid ivgtt.+CoQ tid po	30	Total effectiveness; symptom effectiveness; ECG; myocardial enzyme
Gao and Zhang [47]	38/24	S: 26.4 ± 10.2 W: 25.6 ± 10.4	S: 22/16 W: 11/13	SMS bid + AD + Vit.C bid ivgtt.+CoQ tid po vs AD + Vit.C bid ivgtt.+CoQ tid po	45	Total effectiveness; ECG; cardiac function
Shi [52]	58/58	S: 6.13 ± 1.28 W: 7.52 ± 1.37	S: 27/31 W: 28/30	Ermai Yangxin decoction 1–3/d + ganciclovir + Vit.C&E + gamma globulin + fructose + ATP + fruity potassium vs ganciclovir + Vit.C&E + gamma globulin + fructose + ATP + fruity potassium	NR	Total effectiveness; myocardial enzyme; adverse events
Li and Yan [54]	30/30	16~38	S: 14/16 W: 15/15	Shenqiao Shengmai decoction bid + GIK + penicillin (fosfomycin if allergic) + antiviral drug vs GIK + penicillin (fosfomycin if allergic) + antiviral drug	30	Total effectiveness
Guo and Jin [55]	60/60	16 (6~10: 32, 11~14: 40, 15~23: 48)	64/56	Huangqi SMS bid + AD + inosine qd ivgtt.+ribavirin, propafenone, mexiletine + CoQ tid po vs AD + inosine qd ivgtt.+ribavirin, propafenone, mexiletine + CoQ tid	28	Total effectiveness
Min and Zhang [56]	30/30	18~32	S: 9/21 W: 11/19	SMS bid + corticosteroids (short‐term use) + digitalis and diuretics (heart failure patients) vs corticosteroids (short‐term use) + digitalis and diuretics (heart failure patients)	14	Total effectiveness
Sun H. and Sun P. [57]	50/50	S: <40: 39, >40: 11	S: 20/30		14	Total effectiveness
Patent medicine, 11 RCTs						
Ge [30]	43/43	S: 8.4 ± 2.3 W: 8.2 ± 2.1	S: 23/20 W: 24/19	Huangqi Shengmaiyin tid + creatine phosphate sodium + Vit.C ivgtt. vs creatine phosphate sodium + Vit.C ivgtt.	7	Total effectiveness
Wang et al. [35]	86/85	S: 30.11 ± 6.55 W: 30.58 ± 6.20	S: 39/47 W: 37/48	Lvfukang Capsule 4# tid + GIK bid + Vit.C tid po + CoQ Capsule tid po vs GIK bid + Vit.C tid po + CoQ Capsule tid po	30	Total effectiveness; TCM Syndrome Score; symptom effectiveness; ECG; adverse events
Ren et al. [36]	60/55	S: 23.4 W: 22.7	S: 32/28 W: 30/25	Yixinshu Capsule 3# tid + AD vs AD	14	Total effectiveness; ECG; myocardial enzyme; adverse events
Zhou and Wu [37]	148/148	31.3 ± 7.5	156/140	Yixinshu Capsule 3# tid + GIK + CoQ vs GIK + CoQ	28	Total effectiveness; arrhythmia recovery rate; myocardial enzyme; adverse events
Men [38]	120/120	42 (15~70)	S: 66/54 W: 62/58	Shensong Yangxin Capsule 4# tid + propafenone hydrochloride tid po vs propafenone hydrochloride tid po	180	Total effectiveness; ECG; cardiac function
Zhi [40]	38/30	S: 30.1 (5~47) W: 32.6 (7~48)	S: 17/21 W: 16/14	Shensong Yangxin Capsule 1-4# tid + AD + Vit.C + CoQ vs AD + Vit.C + CoQ	15–30	Total effectiveness
Wang [41]	42/40	S: 34 (15~53) W: 36 (16~55)	S: 25/17 W: 22/18	Shensong Yangxin Capsule 3-4# tid + GIK + Vit.C + 1, 6 fructose diphosphate vs GIK + Vit.C + 1, 6 fructose diphosphate	28	Symptom improvement; ventricular premature beat improvement; adverse events
Li and Huang [48]	49/49	S: 36.14 ± 11.66 W: 37.54 ± 10.85	S: 25/24 W: 27/22	Yixinshu Pill 3#tid + creatine phosphate sodium qd ivgtt.+CoQ Capsule tid vs creatine phosphate sodium qd ivgtt.+CoQ Capsule tid	28	Total effectiveness; myocardial enzyme; inflammatory marks
Cai [49]	19/19	54.3 ± 5.2	21/17	Yixinshu Capsule 3# tid + GIK vs GIK	28	Effectiveness of symptom improvement; myocardial enzyme
He [50]	117/116	S: 8.5 ± 6.7 W: 8.7 ± 6.5	S: 63/54 W: 65/51	Yixinshu Capsule 3# tid + trimetazidine tid po vs trimetazidine tid po	42	Total effectiveness
Hu and Jiang [51]	43/43	S: 38.3 ± 3.2 W: 39.6 ± 3.0	S: 26/17 W: 24/19	Huangqi Shengmaiyin bid + AD + GIK + Vit.C ivgtt. vs AD + GIK + Vit.C ivgtt.	15	Total effectiveness

*SMS + WM vs cWM + WM, 7 RCTs*						
Decoction, 6 RCTs						
Yao [59]	30/30	NR	S: 14/16 W: 12/18	Huangqi Yangxin decoction bid + GIK vs fructose diphosphate 0.5 g tid + Vit.C 0.2 g tid + CoQ tid + GIK	28	Total effectiveness; TCM Syndrome Score; myocardial enzyme
Yu [60]	60/42	S: 26.8 ± 5.7 W: 26.1 ± 5.3	S: 34/26 W: 24/18	Eryin decoction bid + digitalis (heart failure patients) vs AD (Vit.C tid po after 15 days) + CoQ tid po + digitalis (heart failure patients)	28	Total effectiveness; improvement of premature beat, dull heart sound, galloping rhythm, and bradycardia
Zhang, Jin, and Wen [61]	30/30	35.6 ± 3	33/27	Xian SMS tid + antiarrhythic drugs (necessarily) vs CoQ Capsule tid po + antiarrhythic drugs (necessarily)	28	Effectiveness of symptom improvement; ECG; cardiac function; antibody; myocardial enzyme
Li and Wu [62]	166/83	S: 14~25: 63, 26~35: 45, 36~45: 32, 46~55: 21, > 55: 5 W: 14~25: 34, 26~35: 23, 36~45: 14, 46~55: 10, > 55: 2	S: 107/59 W: 53/30	Shengmai Wendan decoction bid + AD + GIK vs CoQ Capsule + Vit.C&E tid po + AD + GIK	90–180	Total effectiveness
Liu [63]	40/40	S: 40.8 ± 4.4 W: 40.3 ± 4.2	S: 22/18 W: 23/17	SMS plus Xuefuzhuyu decoction bid + antiviral therapy vs levocarnitine + antiviral therapy	30	Total effectiveness; adverse events
Zhao [64]	60/60	S: 32.11 ± 5.02 W: 33.04 ± 5.25	S: 34/26 W: 37/23	SMS bid + AD + antiviral therapy + Vit.C ivgtt. vs creatine phosphate sodium + AD + antiviral therapy + Vit.C ivgtt.	28	Total effectiveness; ECG; TCM Syndrome Score; myocardial enzyme; adverse events
Patent medicine, 1 RCT						
Zhang and Sun [58]	42/37	S: 28.6 ± 3.2 W: 29.3 ± 2.8	S: 22/20 W: 24/13	Shensong Yangxin Capsule 4# tid + WM vs amiodarone hydrochloride + WM	28	Ventricular arrhythmia effectiveness; adverse events

*Convalescence (> 6 months) SMS + WM vs WM, 5 trials*						
Decoction, 4 RCTs						
Zhou[27]	25/25	S: 32 ± 4.3 W: 31.4 ± 4.1	S: 15/10 W: 13/12	Huangqi Shengmaiyin bid + AD + GIK + CoQ po vs AD + GIK + CoQ po	56	Total effectiveness; adverse events
Yi, Xu, and Li [31]	30/30	NR	NR	Wenxin Shugan decoction bid + trimetazidine + CoQ vs trimetazidine + CoQ	28	TCM Syndrome Score; HAMD Depression Score; high frequency of heart rate variability
Zhang et al. [43]	42/20	S: 19.4 (15~44) W: 18.2 (15~45)	S: 29/13 W: 13/7	Shengmai Baoyuan decoction tid + AD vs AD	28	Total effectiveness; cardiac function
Ma [53]	20/20	12~56	S: 11/9 W: 10/10	SMS bid + AD + GIK + CoQ tid po vs AD + GIK + CoQ tid po	28	Total effectiveness
Patent medicine, 1 RCT						Total effectiveness
Chen[33]	60/60	S: 35.9 ± 2.9 W: 36.9 ± 1.9	S: 38/22 W: 35/25	Huangqi Shengmaiyin 10–20 ml bid/tid + GIK + Vit.C vs GIK + Vit.C	15	Total effectiveness; myocardial enzyme; complications

*Note:* Total effectiveness is mainly evaluated by symptoms and ECG improvement.

Abbreviations: AD, adenosine disodium; ATP, adenosine triphosphate; bid, twice a day; CoA, coenzyme A; CoQ: coenzyme Q10; ECG, electrocardiogram; F, female; GIK, glucose-insulin-potassium; HAMD, Hamilton Depression Scale; ivgtt., intravenous drip; M, male; NR, not reported; po, take orally; qd, once a day; S, Shengmai San; TCM, traditional Chinese medicine; tid, thrice a day; Vit. C, vitamin C; W, western medicine.

**Table 2:** Summary of effect estimations of SMS for VMC in RCTs.

**Outcomes**	**N**, **n**	**Estimate effect, 95% CI**	**P**
Palpitation disappearance rate			
SMS vs WM	*N* = 2, *n* = 89	RR = 2.3, 1.59-3.33, *I*^2^ = 0%	*P* < 0.00001
SMS + WM vs WM	*N* = 3, *n* = 136	RR = 1.52, 1.21-1.92, *I*^2^ = 0%	*P* = 0.0004
Decoction	*N* = 2, *n* = 162	RR =1.55, 1.20-2.00, *I*^2^ = 0%	*P* = 0.0009
Patent medicine	*N* = 1, *n* = 115	RR = 1.41, 0.82-2.41, *I*^2^ = 0%	*P* = 0.21
Dyspnea disappearance rate			
SMS + WM vs WM	*N* = 3, *n* = 267	RR = 1.47, 1.12-1.94, *I*^2^ = 0%	*P* = 0.006
Decoction	*N* = 2, *n* = 152	RR = 1.50, 1.08-2.06, *I*^2^ = 0%	*P* = 0.01
Patent medicine	*N* = 1, *n* = 115	RR =1.41, 0.82-2.41, *I^2^* = 0%	*P* = 0.21
Chest pain disappearance rate			
SMS vs WM	*N* = 2, *n* = 89	RR = 1.57, 1.17–2.09, *I*^2^ = 0%	*P* = 0.002
SMS + WM vs WM	*N* = 1, *n* = 233	RR = 2.11, 1.38–3.23	*P* = 0.0005
ECG recovery rate			
SMS vs WM	*N* = 3, *n* = 173	RR = 1.33, 0.85–2.09, *I*^2^ = 0%	*P* = 0.21
Decoction	*N* = 2, *n* = 113	RR = 1.48, 0.83–2.65, *I*^2^ = 1%	*P* = 0.18
Patent medicine	*N* = 1, *n* = 60	RR = 1.11, 0.53–2.34	*P* = 0.78
SMS + WM vs WM	*N* = 20, *n* = 2035	RR = 1.46, 1.34–1.59, *I*^2^ = 0%	*P* < 0.00001
Decoction	*N* = 12, *n* = 127	RR = 1.47, 1.31–1.66, *I*^2^ = 0%	*P* < 0.00001
Patent medicine	*N* = 8, *n* = 1008	RR = 1.48, 1.25–1.74, *I*^2^ = 29%	*P* < 0.00001
SMS + WM vs cWM + WM	*N* = 6, *n* = 671	RR = 1.8, 1.28–2.541, *I*^2^ = 61%	*P* = 0.0008
Convalescence-SMS + WM vs WM	*N* = 2, *n* = 160	RR = 1.25, 1.02–1.54, *I*^2^ = 0%	*P* =0.03
Decoction	*N* = 1, *n* = 40	RR = 1.33, 1.57–3.14	*P* =0.51
Patent medicine	*N* = 1, *n* = 120	RR = 1.25, 1.01–1.54	*P* = 0.04
Myocardial injury marker CK-MB			
SMS vs WM	*N* = 1, *n* = 68	MD1299U/L, −3.14–29.12	*P* = 0.11
SMS + WM vs WM	*N* = 8, *n* = 946	MD636U/L, −8.43 to −4.28, *I*^2^ = 94%	*P* < 0.00001
Decoction	*N* = 4, *n* = 399	MD638U/L, −8.93 to −3.84, *I*^2^ = 88%	*P* < 0.00001
Patent medicine	*N* = 4, *n* = 547	MD641U/L, −9.93 to −2.90, *I*^2^ = 96%	*P* = 0.0004
Additional herbs ≤ 6	*N* = 5, *n* = 639	MD565U/L, −8.14 to −3.15, *I*^2^ = 0%	*P* < 0.00001
Additional herbs > 6	*N* = 3, *n* = 307	MD739U/L, −8.54 to −6.24, *I*^2^ = 95%	*P* < 0.00001
SMS + WM vs cWM + WM	*N* = 3, *n* = 240	MD757U/L, −8.83 to −6.32, *I*^2^ = 0%	*P* < 0.00001
Myocardial injury marker cTnI			
SMS vs WM	*N* = 1, *n* = 60	MD082ng/ml, −0.98 to −0.66	*P* < 0.00001
SMS + WM vs WM	*N* = 3, *n* = 307	MD006ng/ml, −0.06 to −0.05, *I*^2^ = 0%	*P* < 0.00001
SMS + WM vs cWM + WM	*N* = 1, *n* = 60	MD067ng/ml, −1.01 to −0.33	*P* = 0.0001
LVEF			
SMS + WM vs WM	*N* = 1, *n* = 62	MD = 8.7%, 4.69-12.71	*P* < 0.0001
SMS + WM vs cWM + WM	*N* = 2, *n* = 180	MD = 5.44%, 1.56–9.33, *I*^2^ = 55%	*P* = 0.006
Convalescence-SMS + WM vs WM	*N* = 1, *n* = 62	MD = 5.85%, 2.16–9.54	*P* = 0.002
The score of general symptoms			
SMS + WM vs WM	*N* = 3, *n* = 449	SMD = −1.11, −1.48 to −0.75, *I*^2^ = 69%	*P* < 0.00001
Decoction	*N* = 2, *n* = 278	SMD = −0.94, −1.24 to −0.63, *I*^2^ = 28%	*P* < 0.00001
Patent medicine	*N* = 1, *n* = 171	SMD = −1.39, −1.73 to −1.06	*P* < 0.00001
SMS + WM vs cWM + WM	*N* = 2, *n* = 180	MD = −7.5, −10.07 to −4.93, *I*^2^ = 87%	*P* < 0.00001
Convalescence-SMS + WM vs WM	N =1, n =60	MD = −6.3, −7.05 to −5.55	*P* < 0.00001
Adverse incidence			
SMS + WM vs WM	*N* = 8, *n* = 1097	RR = 1.22, 0.28−5.39, *I*^2^ = 41.2%	*P* = 0.79
Decoction	*N* = 4, *n* = 433	RR = 0.8, 0.2–3.11, *I*^2^ = 60%	*P* = 0.75
Patent medicine	*N* = 4, *n* = 664	RR = 6.92, 0.36–131.96	*P* = 0.20
SMS + WM vs cWM + WM	*N* = 3, *n* = 279	RR = 0.34, 0.11–1.05, *I*^2^ = 0%	*P* = 0.06
Decoction	*N* = 2, *n* = 200	RR = 0.5, 0.1–2.58	*P* = 0.41
Patent medicine	*N* = 1, *n* = 79	RR = 0.25, 0.06–1.14	*P* = 0.07
Convalescence-SMS + WM vs WM	*N* = 1, *n* = 50	RR = 1.33, 0.33–5.36	*P* = 0.69

Abbreviations: CI, confidence interval; cWM, certain western medicine; MD, mean difference; *n*, number of patients; *N*, number of trials; *P*, probability value; RR, risk ratio; SMD, standard mean difference; SMS, Shengmai San; WM, western medicine.

## Data Availability

No underlying data was collected or produced in this study.

## Supporting Information

Supporting Information Additional supporting information can be found online in the Supporting Information section. Figure S1 Forest plot of subgroup analysis according to dosage forms for ECG recovery rate in comparison of SMS + WM vs WM. Figure S2 Forest plot of subgroup analysis according to additional herbs' quantity for CK-MB in comparison of SMS + WM vs WM. Figure S3 Funnel plot for ECG recovery rate in comparison of SMS + WM vs WM (20 RCTs). Figure S4 Names, property, and function of additional herbs. Table S1 Searching strategies. Table S2 Compositions of SMS in the 44 included trials. Table S3 Adverse events of SMS for VMC in RCTs. Table S4 PRISMA 2020 Checklist.
