# Supplementary material for: Effectiveness and Safety of Shengmai San for Viral Myocarditis: A Systematic Review and Meta-Analysis of Randomized Controlled Trials
Source: Cardiovasc Ther. 2024 Jun 20;2024:2127018. doi: 10.1155/2024/2127018 (PMC11211013; doi:10.1155/2024/2127018)
Supplement: Supporting Information — Additional supporting information can be found online in the Supporting Information section. Figure S1 Forest plot of subgroup analysis according to dosage forms for ECG recovery rate in comparison of SMS + WM vs WM. Figure S2 Forest plot of subgroup analysis according to additional herbs' quantity for CK-MB in comparison of SMS + WM vs WM. Figure S3 Funnel plot for ECG recovery rate in comparison of SMS + WM vs WM (20 RCTs). Figure S4 Names, property, and function of additional herbs. Table S1 Searching strategies. Table S2 Compositions of SMS in the 44 included trials. Table S3 Adverse events of SMS for VMC in RCTs. Table S4 PRISMA 2020 Checklist. [file 2127018.f1.docx]

**Supplementary Table 1** PRISMA 2020 Checklist

| **Section and Topic** | **Item #** | **Checklist item** | **Location where item is reported** |
| --- | --- | --- | --- |
| **TITLE** | | |  |
| Title | 1 | Identify the report as a systematic review. | Page 1 |
| **ABSTRACT** | | |  |
| Abstract | 2 | See the PRISMA 2020 for Abstracts checklist. | Page 1 |
| **INTRODUCTION** | | |  |
| Rationale | 3 | Describe the rationale for the review in the context of existing knowledge. | Page 2 |
| Objectives | 4 | Provide an explicit statement of the objective(s) or question(s) the review addresses. | Page 1-2 |
| **METHODS** | | |  |
| Eligibility criteria | 5 | Specify the inclusion and exclusion criteria for the review and how studies were grouped for the syntheses. | Page 3 |
| Information sources | 6 | Specify all databases, registers, websites, organisations, reference lists and other sources searched or consulted to identify studies. Specify the date when each source was last searched or consulted. | Page 4 |
| Search strategy | 7 | Present the full search strategies for all databases, registers and websites, including any filters and limits used. | Supplementary Table 1 |
| Selection process | 8 | Specify the methods used to decide whether a study met the inclusion criteria of the review, including how many reviewers screened each record and each report retrieved, whether they worked independently, and if applicable, details of automation tools used in the process. | Page 4 |
| Data collection process | 9 | Specify the methods used to collect data from reports, including how many reviewers collected data from each report, whether they worked independently, any processes for obtaining or confirming data from study investigators, and if applicable, details of automation tools used in the process. | Page 4 |
| Data items | 10a | List and define all outcomes for which data were sought. Specify whether all results that were compatible with each outcome domain in each study were sought (e.g. for all measures, time points, analyses), and if not, the methods used to decide which results to collect. | Page 4 |
|  | 10b | List and define all other variables for which data were sought (e.g. participant and intervention characteristics, funding sources). Describe any assumptions made about any missing or unclear information. |  |
| Study risk of bias assessment | 11 | Specify the methods used to assess risk of bias in the included studies, including details of the tool(s) used, how many reviewers assessed each study and whether they worked independently, and if applicable, details of automation tools used in the process. | Page 4 |
| Effect measures | 12 | Specify for each outcome the effect measure(s) (e.g. risk ratio, mean difference) used in the synthesis or presentation of results. | Page 4-5 |
| Synthesis methods | 13a | Describe the processes used to decide which studies were eligible for each synthesis (e.g. tabulating the study intervention characteristics and comparing against the planned groups for each synthesis (item #5)). |  |
|  | 13b | Describe any methods required to prepare the data for presentation or synthesis, such as handling of missing summary statistics, or data conversions. |  |
|  | 13c | Describe any methods used to tabulate or visually display results of individual studies and syntheses. |  |
|  | 13d | Describe any methods used to synthesize results and provide a rationale for the choice(s). If meta-analysis was performed, describe the model(s), method(s) to identify the presence and extent of statistical heterogeneity, and software package(s) used. | Page 4-5 |
|  | 13e | Describe any methods used to explore possible causes of heterogeneity among study results (e.g. subgroup analysis, meta-regression). | Page 5 |
|  | 13f | Describe any sensitivity analyses conducted to assess robustness of the synthesized results. | Page 5 |
| Reporting bias assessment | 14 | Describe any methods used to assess risk of bias due to missing results in a synthesis (arising from reporting biases). | Page 4 |
| Certainty assessment | 15 | Describe any methods used to assess certainty (or confidence) in the body of evidence for an outcome. | Page 5 |
| **RESULTS** | | |  |
| Study selection | 16a | Describe the results of the search and selection process, from the number of records identified in the search to the number of studies included in the review, ideally using a flow diagram. | Page 5-6 |
|  | 16b | Cite studies that might appear to meet the inclusion criteria, but which were excluded, and explain why they were excluded. |  |
| Study characteristics | 17 | Cite each included study and present its characteristics. | Page 7-10 |
| Risk of bias in studies | 18 | Present assessments of risk of bias for each included study. | Page 11 |
| Results of individual studies | 19 | For all outcomes, present, for each study: (a) summary statistics for each group (where appropriate) and (b) an effect estimate and its precision (e.g. confidence/credible interval), ideally using structured tables or plots. | Page 12-14 |
| Results of syntheses | 20a | For each synthesis, briefly summarise the characteristics and risk of bias among contributing studies. |  |
|  | 20b | Present results of all statistical syntheses conducted. If meta-analysis was done, present for each the summary estimate and its precision (e.g. confidence/credible interval) and measures of statistical heterogeneity. If comparing groups, describe the direction of the effect. | Page 12-14 |
|  | 20c | Present results of all investigations of possible causes of heterogeneity among study results. | Page 14 |
|  | 20d | Present results of all sensitivity analyses conducted to assess the robustness of the synthesized results. | Page 14 |
| Reporting biases | 21 | Present assessments of risk of bias due to missing results (arising from reporting biases) for each synthesis assessed. | Page 15 |
| Certainty of evidence | 22 | Present assessments of certainty (or confidence) in the body of evidence for each outcome assessed. |  |
| **DISCUSSION** | | |  |
| Discussion | 23a | Provide a general interpretation of the results in the context of other evidence. | Page 15 |
|  | 23b | Discuss any limitations of the evidence included in the review. | Page 16 |
|  | 23c | Discuss any limitations of the review processes used. |  |
|  | 23d | Discuss implications of the results for practice, policy, and future research. | Page 16 |
| **OTHER INFORMATION** | | |  |
| Registration and protocol | 24a | Provide registration information for the review, including register name and registration number, or state that the review was not registered. | Page 3 |
|  | 24b | Indicate where the review protocol can be accessed, or state that a protocol was not prepared. | Page 3 |
|  | 24c | Describe and explain any amendments to information provided at registration or in the protocol. |  |
| Support | 25 | Describe sources of financial or non-financial support for the review, and the role of the funders or sponsors in the review. | Page 17 |
| Competing interests | 26 | Declare any competing interests of review authors. | Page 17 |
| Availability of data, code and other materials | 27 | Report which of the following are publicly available and where they can be found: template data collection forms; data extracted from included studies; data used for all analyses; analytic code; any other materials used in the review. |  |

**Supplementary Table 2** Searching strategies

**Searching time: June 25, 2023**

| **Database searched** | **Records** |
| --- | --- |
| Chinese National Knowledge Infrastructure Database (CNKI) | 371 |
| Wanfang Database | 346 |
| Chinese Science and Technique Journals Database (VIP) | 79 |
| Chinese Biomedical Literature Database (Sinomed) | 123 |
| Yiigle databases | 1 |
| PubMed | 4 |
| Embase | 6 |
| Cochrane library | 4 |
| **Total** | **934** |

The search strategy takes the English database as examples.

**Pubmed**

(((((((((((((((((((((((((((((((((((((((((((((Shengmai San[Title/Abstract]) OR (Shengmai Decoction[Title/Abstract])) OR (Sheng Mai San[Title/Abstract])) OR (Shengmaisan[Title/Abstract])) OR (Shengmaiyin[Title/Abstract])) OR (Sheng Mai Yin[Title/Abstract])) OR (Dengzhan Shengmai capsule[Title/Abstract])) OR (Dengzhanshengmai capsule[Title/Abstract])) OR (Deng Zhan Sheng Mai capsule[Title/Abstract])) OR (Pingfeng Shengmai capsule[Title/Abstract])) OR (Pingfengshengmai capsule[Title/Abstract])) OR (Ping Feng Sheng Mai capsule[Title/Abstract])) OR (Shengmai Zengyetong Capsule[Title/Abstract])) OR (Shengmaizengyetong Capsule[Title/Abstract])) OR (Sheng Mai Zeng Ye Tong Capsule[Title/Abstract])) OR (Sheng Mai Capsule[Title/Abstract])) OR (Shengmai Capsule[Title/Abstract])) OR (Yangxin Shengmai Granules[Title/Abstract])) OR (Yang Xin Sheng Mai Granules[Title/Abstract])) OR (Yangxinshengmai Granules[Title/Abstract])) OR (Yixin Fumai Granules[Title/Abstract])) OR (Yi Xin Fu Mai Granules[Title/Abstract])) OR (Yixinfumai Granules[Title/Abstract])) OR (Yixin Granules[Title/Abstract])) OR (Yi Xin Granules[Title/Abstract])) OR (Shengmai Granules[Title/Abstract])) OR (Sheng Mai Granules[Title/Abstract])) OR (Shenqifumai[Title/Abstract])) OR (Shen Qi Fu Mai[Title/Abstract])) OR (Yiqifumai[Title/Abstract])) OR (Yi Qi Fu Mai[Title/Abstract])) OR (Yixinshu[Title/Abstract])) OR (Yi Xin Shu[Title/Abstract])) OR (Shensongyangxin Capsule[Title/Abstract])) OR (Shen Song Yang Xin Capsule[Title/Abstract])) OR (Shengmai Tablets[Title/Abstract])) OR (Sheng Mai Tablets[Title/Abstract])) OR (Shengmai Tea Bag[Title/Abstract])) OR (Shengmai Fang[Title/Abstract])) OR (Sheng Mai Fang[Title/Abstract])) OR (Sheng Mai Tang[Title/Abstract])) OR (Shengmai Tang[Title/Abstract])) OR (((((((((((((((Renshen) OR (Ren Shen)) OR (Ginseng)) OR (Panax)) OR (Dangshen)) OR (Dang Shen)) OR (Tangshen)) OR (Codonopsis)) OR (Taizishen)) OR (Taizi Shen)) OR (Tai Zi Shen)) OR (Heterophylly Falsestarwort Root)) OR (False Starwort)) OR (Pseudostellaria heterophylla)) AND ((((Maidong) OR (Mai Dong)) OR (Dwarf Lilyturf)) OR (Ophiopogon japonicus)) AND (((Wuweizi) OR (Wu Wei Zi)) OR (Schisandra))))) AND (myocarditis[Title/Abstract]) ) AND (random*[Title/Abstract])

**Embase**

(('shengmai san':ti,ab,kw OR 'shengmai decoction':ti,ab,kw OR 'sheng mai san':ti,ab,kw OR shengmaisan:ti,ab,kw OR shengmaiyin:ti,ab,kw OR 'sheng mai yin':ti,ab,kw OR 'dengzhan shengmai capsule':ti,ab,kw OR 'dengzhanshengmai capsule':ti,ab,kw OR 'deng zhan sheng mai capsule':ti,ab,kw OR 'pingfeng shengmai capsule':ti,ab,kw OR 'pingfengshengmai capsule':ti,ab,kw OR 'ping feng sheng mai capsule':ti,ab,kw OR 'shengmai zengyetong capsule':ti,ab,kw OR 'shengmaizengyetong capsule':ti,ab,kw OR 'sheng mai zeng ye tong capsule':ti,ab,kw OR 'sheng mai capsule':ti,ab,kw OR 'shengmai capsule':ti,ab,kw OR 'yangxin shengmai granules':ti,ab,kw OR 'yang xin sheng mai granules':ti,ab,kw OR 'yangxinshengmai granules':ti,ab,kw OR 'yixin fumai granules':ti,ab,kw OR 'yi xin fu mai granules':ti,ab,kw OR 'yixinfumai granules':ti,ab,kw OR 'yixin granules':ti,ab,kw OR 'yi xin granules':ti,ab,kw OR 'shengmai granules':ti,ab,kw OR 'sheng mai granules':ti,ab,kw OR shenqifumai:ti,ab,kw OR 'shen qi fu mai':ti,ab,kw OR yiqifumai:ti,ab,kw OR 'yi qi fu mai':ti,ab,kw OR yixinshu:ti,ab,kw OR 'yi xin shu':ti,ab,kw OR 'shensongyangxin capsule':ti,ab,kw OR 'shen song yang xin capsule':ti,ab,kw OR 'shengmai tablets':ti,ab,kw OR 'sheng mai tablets':ti,ab,kw OR 'shengmai tea bag':ti,ab,kw OR 'shengmai fang':ti,ab,kw OR 'sheng mai fang':ti,ab,kw OR 'sheng mai tang':ti,ab,kw OR 'shengmai tang':ti,ab,kw) OR ((renshen:ti,ab,kw OR 'ren shen':ti,ab,kw OR ginseng:ti,ab,kw OR panax:ti,ab,kw OR dangshen:ti,ab,kw OR 'dang shen':ti,ab,kw OR tangshen:ti,ab,kw OR codonopsis:ti,ab,kw OR taizishen:ti,ab,kw OR 'taizi shen':ti,ab,kw OR 'tai zi shen':ti,ab,kw OR 'heterophylly falsestarwort root':ti,ab,kw OR 'false starwort':ti,ab,kw OR 'pseudostellaria heterophylla':ti,ab,kw) AND (maidong:ti,ab,kw OR 'mai dong':ti,ab,kw OR 'dwarf lilyturf':ti,ab,kw OR 'ophiopogon japonicus':ti,ab,kw) AND (wuweizi:ti,ab,kw OR 'wu wei zi':ti,ab,kw OR schisandra:ti,ab,kw))) AND (random*:ti,ab,kw AND myocarditis:ti,ab,kw)

**Cochrane library**

#1 myocarditis

#2 Shengmai San

#3 Shengmai Decoction

#4 Sheng Mai San

#5 Shengmaisan

#6 Renshen

#7 Ren Shen

#8 Ginseng

#9 Panax

#10 Dangshen

#11 Dang Shen

#12 Tangshen

#13 Codonopsis

#14 Taizishen

#15 Taizi Shen

#16 Tai Zi Shen

#17 Heterophylly Falsestarwort Root

#18 False Starwort

#19 Pseudostellaria heterophylla

#20 #6 OR #7 OR #8 OR #9 OR #10 OR #11 OR #12 OR #13 OR #14 OR #15 OR #16 OR #17 OR #18 OR #19

#21 Maidong

#22 Mai Dong

#23 Dwarf Lilyturf

#24 Ophiopogon japonicus

#25 #21 OR #22 OR #23 OR #24

#26 Wuweizi

#27 Wu Wei Zi

#28 Schisandra

#29 #26 OR #27 OR #28

#30 #20 AND #25 AND #29

#31 Shengmaiyin

#32 Sheng Mai Yin

#33 Dengzhan Shengmai capsule

#34 Dengzhanshengmai capsule

#35 Deng Zhan Sheng Mai capsule

#36 Pingfeng Shengmai capsule

#37 Pingfengshengmai capsule

#38 Ping Feng Sheng Mai capsule

#39 Shengmai Zengyetong Capsule

#40 Shengmaizengyetong Capsule

#41 Sheng Mai Zeng Ye Tong Capsule

#42 Sheng Mai Capsule

#43 Shengmai Capsule

#44 Yangxin Shengmai Granules

#45 Yang Xin Sheng Mai Granules

#46 Yangxinshengmai Granules

#47 Yixin Fumai Granules

#48 Yi Xin Fu Mai Granules

#49 Yixinfumai Granules

#50 Yixin Granules

#51 Yi Xin Granules

#52 Shengmai Granules

#53 Sheng Mai Granules

#54 Shenqifumai

#55 Shen Qi Fu Mai

#56 Yiqifumai

#57 Yi Qi Fu Mai

#58 Yixinshu

#59 Yi Xin Shu

#60 Shensongyangxin Capsule

#61 Shen Song Yang Xin Capsule

#62 Shengmai Tablets

#63 Sheng Mai Tablets

#64 Shengmai Tea Bag

#65 Shengmai Fang

#66 Sheng Mai Fang

#67 Sheng Mai Tang

#68 Shengmai Tang

#69 #2 OR #3 OR #4 OR #5 OR #30 OR #31 OR #32 OR #33 OR #34 OR #35 OR #36 OR #37 OR #38 OR #39 OR #40 OR #41 OR #42 OR #43 OR #44 OR #45 OR #46 OR #47 OR #48 OR #49 OR #50 OR #51 OR #52 OR #53 OR #54 OR #55 OR #56 OR #57 OR #58 OR #59 OR #60 OR #61 OR #62 OR #63 OR #64 OR #65 OR #66 OR #67 OR #68

#70 random*

#71 #1 AND #69 AND #70

**Supplementary Table 3** Compositions of SMS in the 44 included trials

| StudyID | Name of SMS | Compositions |
| --- | --- | --- |
| Decoctions | |  |
| ZhangLP 2017 | Shengmai Xianxiong decoction△ | Panax ginseng C.A.Mey. 10g, Ophiopogon japonicus (Thunb.) Ker Gawl. 15g, Schisandra chinensis (Turcz.) Baill. 3g, Coptis chinensis Franch. 5g, Pinellia ternata (Thunb.) Makino 10g, Trichosanthes kirilowii Maxim. 15g, Citrus aurantium L. 10g, Salvia miltiorrhiza Bunge 15g, Curcuma rubescens Roxb. 10g, Acorus gramineus Aiton 10g, Viola maculata Cav. 15g. |
| LiJ 2005 | Qingxin Shengmai decoction | Coptis chinensis Franch. 10g, Sophora velutina Lindl. 10g, Codonopsis pilosula Nannf. 15g, Ophiopogon japonicus (Thunb.) Ker Gawl. 15g, Schisandra chinensis (Turcz.) Baill. 5g, Glehnia littoralis F.Schmidt 15g, Scrophularia kakudensis Franch. 12g, Salvia miltiorrhiza Bunge 30g, Curcuma rubescens Roxb. 10g, Ziziphus jujuba var. spinosa (Bunge) Hu ex H.F.Chow. 15g, Cinnamomum verum J.Presl 6g, Glycyrrhiza uralensis Fisch. ex DC. (honey-fried) 10g.Continuing low-heating, add Vincetoxicum atratum (Bunge) C.Morren & Decne 10g, Lycium chinense Mill. 15g; Qi-stagnancy and blood stasis, add Paeonia suffruticosa Andrews 10g; Prunus persica(L.)Batsch 30g, tachycardia, add Os Draconis 30g, Concha Ostreae 30gy, Polygala tenuifolia Willd. 10g; yellowish fur, add Bambusa tuldoides Munro 10g, Citrus reticulata Blanco 6g; red and dry tongue, add Rehmannia Libosch. ex Fisch. & C.A.Mey. 12g, Polygonatum odoratum (Mill.) Druce 10g; fat and pale tongue, add Rheum palmatum f. rubiflora Stapf 30g. |
| ZhouGM 2005 | Shengmai Huangqiguizhi decoction△ | Pseudostellaria heterophylla (Miq.) Pax, Ophiopogon japonicus (Thunb.) Ker Gawl., Ziziphus jujuba Mill., Ziziphus jujuba Mill. 15g each, Schisandra chinensis (Turcz.) Baill., Zingiber officinale Roscoe 8g, Eleutherococcus henryi Oliv., Salvia miltiorrhiza Bunge 30g, Cinnamomum verum J.Presl 6g, Paeonia lactiflora Pall 12g, Glycyrrhiza uralensis Fisch. ex DC. (honey-fried) 10g. Excessive noxious heat, add Coptis chinensis Franch. 6g, Coptis chinensis Franch. 15g, Scrophularia kakudensis Franch. 15g; Qi-stagnancy and blood stasis, add Paeonia suffruticosa Andrews 12g, Prunus persica(L.)Batsch 10g; premature beat frequently, add Sophora velutina Lindl. 15g; tachycardia, add Os Draconis 30g, Concha Ostreae 30g, Polygala tenuifolia Willd. 10g. |
| LiSW 2019 | Huangqi Shengmai Yin△ | Codonopsis pilosula Nannf. 20g, Schisandra chinensis (Turcz.) Baill. 20g, Eleutherococcus henryi Oliv. 30-60g, Ophiopogon japonicus (Thunb.) Ker Gawl. 25g. Qi-stagnancy and blood stasis, add Curcuma rubescens Roxb. 15g, Conioselinum Chuanxiong 15g; sleep disorders, add Platycladus orientalis (L.) Franco 20g, Wolfiporia cocos (F.A. Wolf) Ryvarden & Gilb. 15g, Ziziphus jujuba var. spinosa (Bunge) Hu ex H.F.Chow. 15g, Polygala tenuifolia Willd. 10g; phlegm syndrome, add Atractylodes macrocephala Koidz 16g, Wolfiporia cocos (F.A. Wolf) Ryvarden & Gilb. 16g. |
| YaoY 2019 | Shengmai San plus Xiaochaihu decoction | Bupleurum chinense DC. 12g, Scutellaria baicalensis Georgi, Pinellia ternata (Thunb.) Makino, Ophiopogon japonicus (Thunb.) Ker Gawl., Zingiber officinale Roscoe 9g each, Schisandra chinensis (Turcz.) Baill. 6g, Panax ginseng C.A.Mey. 6g, Glycyrrhiza uralensis Fisch. ex DC. 5g, Ziziphus jujuba Mill. 4 dates. Severe palpitation, add Salvia miltiorrhiza Bunge 15g, Semen Zizyphi Spinosae 15g; Severe chest tightness, add Curcuma rubescens Roxb. 15g, Inula japonica Thunb. 10g; phlegm syndrome and Xiongbi, add Allium macrostemon Bunge 10g, Trichosanthes kirilowii Maxim. 10g. |

**Supplementary Table 3** Compositions of SMS in the 44 included trials (Continued)

| StudyID | Name of SMS | Compositions |
| --- | --- | --- |
| TanSW 2018，LiYL 2015 | Ermai Yangxin decoction1 | Pleuropterus multiflorus Turcz. ex Nakai 15g, Pseudostellaria heterophylla (Miq.) Pax, Ophiopogon japonicus (Thunb.) Ker Gawl., Salvia miltiorrhiza Bunge, Scrophularia kakudensis Franch., Paeonia lactiflora Pall, Cannabis sativa L., Ligustrum lucidum W.T.Aiton, Platycladus orientalis (L.) Franco, Eleutherococcus henryi Oliv., Chrysanthemum indicum L., Forsythia suspensa (Thunb.) Vahl 10g each, Schisandra chinensis (Turcz.) Baill., Cinnamomum verum J.Presl, Acorus gramineus Aiton 6g each, Glycyrrhiza uralensis Fisch. ex DC. (honey-fried) 4g. |
| YiL 2015 | Wenxin Shugan decoction | Eleutherococcus henryi Oliv. 30g, Panax ginseng C.A.Mey., Ophiopogon japonicus (Thunb.) Ker Gawl., Schisandra chinensis (Turcz.) Baill., Citrus aurantium L., Cyperus rotundus L., Citrus reticulata Blanco, Ziziphus jujuba var. spinosa (Bunge) Hu ex H.F.Chow., Sophora velutina Lindl. 10g each, Bupleurum chinense DC., Wolfiporia cocos (F.A. Wolf) Ryvarden & Gilb., Polygala tenuifolia Willd., Glycyrrhiza uralensis Fisch. ex DC. (honey-fried) 12g each. |
| ShengAM 2014 | Shengmai San1 | Codonopsis pilosula Nannf. 30g, Ophiopogon japonicus (Thunb.) Ker Gawl. 15g, Schisandra chinensis (Turcz.) Baill. 10g. Wind heat syndrome, add Lonicera japonica Thunb. 15g, Forsythia suspensa (Thunb.) Vahl 15g; blood stasis, add Salvia miltiorrhiza Bunge 15g, Paeonia veitchii Lynch 15g; deficiency of heart qi, add Eleutherococcus henryi Oliv. 20g, Glycyrrhiza uralensis Fisch. ex DC. (honey-fried) 15g; yin-yang deficiency, add Cinnamomum verum J.Presl 15g, Glycyrrhiza uralensis Fisch. ex DC.(honey-fried) 15g. |
| ZhouBL 2011 | Shengmai San△1 | Ophiopogon japonicus (Thunb.) Ker Gawl., Schisandra chinensis (Turcz.) Baill., Codonopsis pilosula Nannf. 6g each. Chest tightness, add Citrus aurantium L. 6g, Conioselinum Chuanxiong 6g, Trichosanthes kirilowii Maxim. 3g, Allium macrostemon Bunge 5g; palpitation, add Ziziphus jujuba var. spinosa (Bunge) Hu ex H.F.Chow. 6g; dark purple tongue, add Prunus persica(L.)Batsch 3g, Carthamus tinctorius L. 5g; chest pain, add Salvia miltiorrhiza Bunge 6g, Paeonia veitchii Lynch 6g; fever, add Lonicera japonica Thunb. 10g, Forsythia suspensa (Thunb.) Vahl, Isatis tinctoria L., Bupleurum chinense DC. 6g; coughing, add Prunus armeniaca L., Fritillaria thunbergii Miq., Tussilago farfara L., Aster tataricus L.f. 6g each. |
| ZhuL 2008 | Shengmai Sanhuangjiedu decoction | Codonopsis pilosula Nannf. 6g, Ophiopogon japonicus (Thunb.) Ker Gawl. 6g, Schisandra chinensis (Turcz.) Baill. 5g, Cornus officinalis var. koreana Kitam. 5g, Nelumbo nucifera Gaertn. 1g, Gardenia jasminoides J. Ellis 2g, Scutellaria baicalensis Georgi, Coptis chinensis Franch., Phellodendron chinense var. glabriusculum C.K.Schneid. 5g each, Isatis tinctoria L. 8g, Lonicera japonica Thunb. 12g, Forsythia suspensa (Thunb.) Vahl 10g, Salvia miltiorrhiza Bunge 10g, Citrus reticulata Blanco 6g, Glycyrrhiza uralensis Fisch. ex DC. 4g. Chest stuffiness, add Trichosanthes kirilowii Maxim. 8g; dysphoria, add Bambusa tuldoides Munro 3g; exuberant fire and heat, add Gypsum Fibrosum 15g; yin deficiency, add Rehmannia Libosch. ex Fisch. & C.A.Mey. 5g; tachycardia, add Sophora velutina Lindl. 6g; bradycardia,add Cinnamomum verum J.Presl 6g. |

**Supplementary Table 3** Compositions of SMS in the 44 included trials (Continued)

| StudyID | Name of SMS | Compositions |
| --- | --- | --- |
| Zhang  JX 2008 | Shengmai Baoyuan decoction | Panax quinquefolius L. 15g, Eleutherococcus henryi Oliv. 30g, Ophiopogon japonicus (Thunb.) Ker Gawl. 15g, Rehmannia Libosch. ex Fisch. & C.A.Mey. 24g, FSchisandra chinensis (Turcz.) Baill. 10g, Coptis chinensis Franch. 6g, Conioselinum Chuanxiong 15g, Paeonia veitchii Lynch 12g, Glycyrrhiza uralensis Fisch. ex DC. 6g. |
| SuYX 2007 | Qilian Shengmai Yin | Eleutherococcus henryi Oliv. 30g, Coptis chinensis Franch. 9g, Panax ginseng C.A.Mey. 9g, Polygonatum sibiricum Redouté 12g, Ophiopogon japonicus (Thunb.) Ker Gawl. 15g, Schisandra chinensis (Turcz.) Baill. 9g, Salvia miltiorrhiza Bunge 20g, Angelica sinensis (Oliv.) Diels 12g, Conioselinum Chuanxiong 12g, Ziziphus jujuba var. spinosa (Bunge) Hu ex H.F.Chow. 30g, Platycladus orientalis (L.) Franco 20g, Glycyrrhiza uralensis Fisch. ex DC. (honey-fried) 12g. |
| TaoH 2003 | Shengmai San plus Zhigancao decoction△ | Eleutherococcus henryi Oliv. 30g, Radix Panacis Ginseng 10g, Ophiopogon japonicus (Thunb.) Ker Gawl. 15g, Schisandra chinensis (Turcz.) Baill. 10g, Glycyrrhiza uralensis Fisch. ex DC. (honey-fried) 12g, Rehmannia Libosch. ex Fisch. & C.A.Mey. 15g, Salvia miltiorrhiza Bunge 15g, Isatis tinctoria L. 30g, Forsythia suspensa (Thunb.) Vahl 15g, Sophora velutina Lindl. 30g, Panax notoginseng (Burkill) F.H.Chen (swallowed) 5g. |
| SuDS 2001 | Shengmai Yuxin decoction | Panax ginseng C.A.Mey. 10g, Ophiopogon japonicus (Thunb.) Ker Gawl. 15g, Schisandra chinensis (Turcz.) Baill. 6g, Eleutherococcus henryi Oliv. 30g, Polygonatum sibiricum Redouté, Rehmannia Libosch. ex Fisch. & C.A.Mey., Dioscorea polystachya Turcz. 15g each, Salvia miltiorrhiza Bunge 24g, Lonicera japonica Thunb. 24g, Conioselinum Chuanxiong, Isatis tinctoria L., Forsythia suspensa (Thunb.) Vahl 12g each. Chest pain, add Panax notoginseng (Burkill) F.H.Chen 3g, Carthamus tinctorius L. 10g; severe palpitation, add Sophora velutina Lindl. 18g, Platycladus orientalis (L.) Franco 12g; severe chest tightness, add Trichosanthes kirilowii Maxim. 15g. |
| GaoZF 1999 | Shengmai San2 | Panax ginseng C.A.Mey. 10g, Ophiopogon japonicus (Thunb.) Ker Gawl. 12g, Schisandra chinensis (Turcz.) Baill. 6g, Eleutherococcus henryi Oliv. 30g, Scrophularia kakudensis Franch., Rehmannia Libosch. ex Fisch. & C.A.Mey., Forsythia suspensa (Thunb.) Vahl, Isatis tinctoria L., Salvia miltiorrhiza Bunge, Rosa rugosa Thunb. 15g each, Lonicera japonica Thunb. 30g, Isatis tinctoria L. 12g, Carthamus tinctorius L. 12g. Severe chest tightness, add Trichosanthes kirilowii Maxim. 15g; chest pain, add Panax notoginseng (Burkill) F.H.Chen 3g, Curcuma rubescens Roxb. 12g; insomnia and dreaminess, add Ziziphus jujuba var. spinosa (Bunge) Hu ex H.F.Chow. 30g, Albizia julibrissin var. julibrissin 15g; cold limbs, subtract Rehmannia Libosch. ex Fisch. & C.A.Mey. and Scrophularia kakudensis Franch., add Aconitum vulparia Rchb. 10g, Zingiber officinale Roscoe 6g. |
| LinSJ 2023 | Yangxin decoction | Panax ginseng C.A.Mey., Ophiopogon japonicus (Thunb.) Ker Gawl., Eleutherococcus henryi Oliv., Glycyrrhiza uralensis Fisch. ex DC. (honey-fried) 20g each, Schisandra chinensis (Turcz.) Baill., Salvia miltiorrhiza Bunge, Ziziphus jujuba var. spinosa (Bunge) Hu ex H.F.Chow., Conioselinum Chuanxiong 15g each, Cornus officinalis var. koreana Kitam. 10g, Nardostachys jatamansi (D. Don) DC. 5g. |

**Supplementary Table 3** Compositions of SMS in the 44 included trials (Continued)

| StudyID | Name of SMS | | Compositions |
| --- | --- | --- | --- |
| ShiH 2017 | Ermai Yangxin decoction2 | Glycyrrhiza uralensis Fisch. ex DC. (honey-fried) 8g, Chrysanthemum indicum L. 8g, Pleuropterus multiflorus Turcz. ex Nakai, Schisandra chinensis (Turcz.) Baill., Acorus gramineus Aiton, Ziziphus jujuba var. spinosa (Bunge) Hu ex H.F.Chow. 10g each, Platycladus orientalis (L.) Franco, Ligustrum lucidum W.T.Aiton, Paeonia veitchii Lynch, Atractylodes macrocephala Koidz, Forsythia suspensa (Thunb.) Vahl 12g each, Angelica sinensis (Oliv.) Diels, Codonopsis pilosula Nannf., Rehmannia Libosch. ex Fisch. & C.A.Mey., Wolfiporia cocos (F.A. Wolf) Ryvarden & Gilb. 15g each, Panax ginseng C.A.Mey., Eleutherococcus henryi Oliv., Rehmannia Libosch. ex Fisch. & C.A.Mey. 25g. Chest tightness, add Bupleurum chinense DC., Paeonia lactiflora Pall 10g each; the weak, add Ziziphus jujuba Mill., Asarum heterotropoides F. Schmidt, Cinnamomum verum J.Presl 10g each; expectoration, add Pinellia ternata (Thunb.) Makino, Trichosanthes kirilowii Maxim., Wolfiporia cocos (F.A. Wolf) Ryvarden & Gilb. 10g each. | |
| MaXN 2010 | Shengmai San△2 | Codonopsis pilosula Nannf. 15g, Ophiopogon japonicus (Thunb.) Ker Gawl. 15g, Eleutherococcus henryi Oliv. 30g, Schisandra chinensis (Turcz.) Baill. 15g, Salvia miltiorrhiza Bunge 20g, Angelica sinensis (Oliv.) Diels 15g, Conioselinum Chuanxiong 15g, Forsythia suspensa (Thunb.) Vahl 20g, Lonicera japonica Thunb. 15g. Myocardial enzyme increase, add Isatis tinctoria L. 15g, Curcuma rubescens Roxb. 15g; arrhythmia, add Corydalis yanhusuo (Y. H. Chou & C. C. Hsu) W. T. Wang ex Z. Y. Su & C. Y. Wu 15g, Crataegus pinnatifida Bunge 10g. | |
| LiRH 2007 | Shenqiao Shengmai decoction | Salvia miltiorrhiza Bunge 18g, Forsythia suspensa (Thunb.) Vahl 10g, Codonopsis pilosula Nannf. 15g, Ophiopogon japonicus (Thunb.) Ker Gawl. 12g, Schisandra chinensis (Turcz.) Baill. 15g, Cornus officinalis var. koreana Kitam. 30g, Nelumbo nucifera Gaertn. 1g, Glycyrrhiza uralensis Fisch. ex DC. 6g. Chest tightness, add Trichosanthes kirilowii Maxim. 18g, Allium macrostemon Bunge 6g; restlessness, add Bambusa tuldoides Munro 15g, Gardenia jasminoides J. Ellis 6g; exuberant fire and heat, add Gypsum Fibrosum 30g, Scutellaria baicalensis Georgi 10g; yin deficiency, add Rehmannia Libosch. ex Fisch. & C.A.Mey. 20g, Scrophularia kakudensis Franch. 15g; tachycardia, add Sophora velutina Lindl. 30g, Magnetitum 20g; bradycardia, add Cinnamomum verum J.Presl 10g; premature beat, add Os Draconis, Concha Ostreae 30g. | |
| GuoYH 2011 | Huangqi Shengmai San△ | Eleutherococcus henryi Oliv. 10-30g, Codonopsis pilosula Nannf. 10-30g, Ophiopogon japonicus (Thunb.) Ker Gawl. 6-10g, Schisandra chinensis (Turcz.) Baill., Salvia miltiorrhiza Bunge, Conioselinum Chuanxiong, Paeonia veitchii Lynch, Carthamus tinctorius L. 6-15g each, Isatis tinctoria L. 10-30g, Lonicera japonica Thunb. 10-20g, Glycyrrhiza uralensis Fisch. ex DC. (honey-fried) 3-10g. | |
| MinJ 2007 | Shengmai San△3 | Panax ginseng C.A.Mey. (decocted separately) 9g, Eleutherococcus henryi Oliv. 30g, Ophiopogon japonicus (Thunb.) Ker Gawl. 9g, Schisandra chinensis (Turcz.) Baill. 6g, Schisandra chinensis (Turcz.) Baill. 20g, Trichosanthes kirilowii Maxim. 15g, Allium macrostemon Bunge 10g, Polygonatum odoratum (Mill.) Druce 12g, Ziziphus jujuba var. spinosa (Bunge) Hu ex H.F.Chow. 12g, Wolfiporia cocos (F.A. Wolf) Ryvarden & Gilb. 24g, Succinum 6g, Glycyrrhiza uralensis Fisch. ex DC. (honey-fried) 6g. | |

**Supplementary Table 3** Compositions of SMS in the 44 included trials (Continued)

| StudyID | Name of SMS | Compositions |
| --- | --- | --- |
| SunH 2007 | Shengmai San△4 | Panax ginseng C.A.Mey. (decocted separately) 12g, Eleutherococcus henryi Oliv. 20g, Ophiopogon japonicus (Thunb.) Ker Gawl. 9g, Schisandra chinensis (Turcz.) Baill. 6g, Schisandra chinensis (Turcz.) Baill. 20g, Polygonatum odoratum (Mill.) Druce, Ziziphus jujuba var. spinosa (Bunge) Hu ex H.F.Chow. 15g, Wolfiporia cocos (F.A. Wolf) Ryvarden & Gilb. 25g, Glycyrrhiza uralensis Fisch. ex DC. (honey-fried) 10g. |
| YaoH 2010 | Huangqi Yanxin decoction | Panax ginseng C.A.Mey. (decocted separately) 5g, Eleutherococcus henryi Oliv. 20g, Ophiopogon japonicus (Thunb.) Ker Gawl. 10g, Schisandra chinensis (Turcz.) Baill. 5g, Schisandra chinensis (Turcz.) Baill., Rehmannia Libosch. ex Fisch. & C.A.Mey. 20g, Conioselinum Chuanxiong 10g, Polygala tenuifolia Willd. 10g, Ziziphus jujuba var. spinosa (Bunge) Hu ex H.F.Chow. 10, Wolfiporia cocos (F.A. Wolf) Ryvarden & Gilb. 20g, Glycyrrhiza uralensis Fisch. ex DC. (honey-fried) 6g. |
| YuSY 2005 | Eryin decoction | Lonicera japonica Thunb. 10g, Taraxacum stevenii DC., Chrysanthemum indicum L., Viola maculata Cav. 30g each, Pseudostellaria heterophylla (Miq.) Pax, Salvia miltiorrhiza Bunge, Wolfiporia cocos (F.A. Wolf) Ryvarden & Gilb., Ophiopogon japonicus (Thunb.) Ker Gawl., Schisandra chinensis (Turcz.) Baill., Cinnamomum verum J.Presl 15g each, Glycyrrhiza uralensis Fisch. ex DC. 6g. |
| ZhangGF 2003 | Xian Shengmai San | Epimedium brevicornu Maxim. 3g, Codonopsis pilosula Nannf. 5g, Ophiopogon japonicus (Thunb.) Ker Gawl. 5g, Schisandra chinensis (Turcz.) Baill. 2g. |
| LiY 2003 | Shengmai Wendan decoction | Panax ginseng C.A.Mey. 10g, Ophiopogon japonicus (Thunb.) Ker Gawl. 15g, Schisandra chinensis (Turcz.) Baill. 6g, Pinellia ternata (Thunb.) Makino 10g, Citrus aurantium L. 10g, Bambusa tuldoides Munro 12g, Citrus reticulata Blanco 10g, Wolfiporia cocos (F.A. Wolf) Ryvarden & Gilb. 10g, Eleutherococcus henryi Oliv. 60g, Sophora velutina Lindl. 10g, Glycyrrhiza uralensis Fisch. ex DC. 10g, Ziziphus jujuba Mill. 10 dates. |
| LiuJL 2016 | Shengmaiyin plus Xuefuzhuyu decoction△ | Ophiopogon japonicus (Thunb.) Ker Gawl., Prunus persica(L.)Batsch, Schisandra chinensis (Turcz.) Baill., Angelica sinensis (Oliv.) Diels, Carthamus tinctorius L., Rehmannia Libosch. ex Fisch. & C.A.Mey., Conioselinum Chuanxiong, Platycodon grandiflorus A.DC., Paeonia veitchii Lynch, Achyranthes bidentata Blume, Citrus aurantium L., Forsythia suspensa (Thunb.) Vahl, Dryopteris crassirhizoma Nakai 10g each, Pseudostellaria heterophylla (Miq.) Pax 15g, Eleutherococcus henryi Oliv. 20g. |
| ZhaoDY 2015 | Shengmai San△5 | Eleutherococcus henryi Oliv. 20g, Codonopsis pilosula Nannf. 20g, Salvia miltiorrhiza Bunge, Schisandra chinensis (Turcz.) Baill., Paeonia veitchii Lynch, Conioselinum Chuanxiong, Panax ginseng C.A.Mey., Lonicera japonica Thunb., Isatis tinctoria L. 10g each, Carthamus tinctorius L. 6g, Glycyrrhiza uralensis Fisch. ex DC. (honey-fried) 3g. Pyrexia, add Bupleurum chinense DC. 6g; dizziness and fatigue, add Lycium chinense Mill. 6g; sore throat, add Arctium lappa L. 5g; palpitation and lower limb edema, add Os Draconis 5g, Succinum 5g; pericardial effusion, add Ziziphus jujuba Mill. 3g, Draba nemorosa L. 4g. |

**Supplementary Table 3** Compositions of SMS in the 44 included trials (Continued)

| StudyID | Name of SMS | Compositions |
| --- | --- | --- |
| Patent medicine | |  |
| XuDG 2015 | Rongxin Wan plus Shensong Yangxin Capsule | Rongxin Wan: Polygonatum odoratum (Mill.) Druce, Salvia miltiorrhiza Bunge, Schisandra chinensis (Turcz.) Baill., Dalbergia odoriferaT. Chen, Sophora velutina Lindl., Isatis tinctoria L. |
| LiuHJ 2010，  MenXY 2012，  ZhiLQ 2009，  WangXH 2009，ZhangDM 2011 | Shensong Yangxin Capsule | Panax ginseng C.A.Mey., Ophiopogon japonicus (Thunb.) Ker Gawl., Cornus officinalis var. koreana Kitam., Salvia miltiorrhiza Bunge, Ziziphus jujuba var. spinosa (Bunge) Hu ex H.F.Chow., Taxillus sutchuenensis Danser, Paeonia veitchii Lynch, Eupolyphaga Seu Opistholpatia, Nardostachys jatamansi (D. Don) DC., Coptis chinensis Franch., Schisandra chinensis (Turcz.) Baill., Os Draconis. |
| GePC 2016，ChenBY 2014，HuXF 2015 | Huangqi Shengmaiyin | Codonopsis pilosula Nannf., Ophiopogon japonicus (Thunb.) Ker Gawl., Schisandra chinensis (Turcz.) Baill., Eleutherococcus henryi Oliv. |
| WangZT 2014 | Lvfukang Capsule | Ophiopogon japonicus (Thunb.) Ker Gawl., Schisandra chinensis (Turcz.) Baill., Panax ginseng C.A.Mey., Salvia miltiorrhiza Bunge, Glehnia littoralis F.Schmidt, Dimocarpus longan Lour., Ziziphus jujuba var. spinosa (Bunge) Hu ex H.F.Chow., Platycladus orientalis (L.) Franco, Os Draconis, Concha Ostreae, Pleuropterus multiflorus Turcz. ex Nakai, Cornus officinalis var. koreana Kitam., Conioselinum Chuanxiong. |
| RenFW 2014，ZhouY 2013，CaiK 2018，HeJB 2015 | Yixinshu Capsule | Ophiopogon japonicus (Thunb.) Ker Gawl., Schisandra chinensis (Turcz.) Baill., Panax ginseng C.A.Mey., Eleutherococcus henryi Oliv., Salvia miltiorrhiza Bunge, Conioselinum Chuanxiong, Crataegus pinnatifida Bunge. |
| LiWT 2022 | Yixinshu Pill |  |

“△” means compositions included additional herbs based on Shengmai San.

**Supplementary Table 4** Adverse events of SMS for VMC in RCTs

| Study ID | Sample size test/control | Incidence | Test Group | Control Group |
| --- | --- | --- | --- | --- |
| S vs W | | |  |  |
| XuDG 2015 | 60 30/30 | 0 | None | None |
| LiuHJ 2010 | 68 34/34 | S: 0  W: NR | None | NR |
| S + W vs W | | | | |
| LiSW 2019 | 50 25/25 | S: 4/25  W: 3/25 | 2 for inappetence; 1 for dizziness and headache;  1 for diarrhea | 1 for inappetence;  2 for dizziness and headache |
| YaoY 2019 | 92 46/46 | S: 4/46  W: 2/46 | 2 for nausea;  2 for anemia | 1 for inappetence;  1 for nausea |
| LiYL 2015 | 116 55/56 | 0 | None for abnormal  of ECG, BUS Rt, hepatic and renal function | None for abnormal  of ECG, BUS Rt, hepatic and renal function |
| WangZT 2014 | 176 86/85 | S: 3/86  W: 0/85 | 3 for diarrhea (disappeared after medication and diet adjustment) | None for abnormal  of BUS Rt, hepatic and renal function etc. |
| RenFW 2014 | 115 60/55 | 0 | None for abnormal  of BUS Rt, hepatic and renal function etc. | None for abnormal  of BUS Rt, hepatic and renal function etc. |
| ZhouY 2013 | 296 148/148 | 0 | None | None |
| ZhouBL 2011 | 114 56/58 | 0 | None | None |
| WangXH 2009 | 82 42/40 | 0 | None for abnormal  of Blood Rt, hepatic and renal function, electrolyte etc. | None for abnormal  of Blood Rt, hepatic and renal function, electrolyte etc. |
| ShiH 2017 | 116 58/58 | S: 9/58  W: 19/58 | NR | NR |
| S + W vs cWM + W | | | | |
| ZhangDM 2011 | 79 42/37 | S: 2/42  W: 7/37 | 2 for inappetence (tolerable) | 2 for sinus arrest; 3 for hepatic function damage; 1 for nausea and vomiting; 1 for thyroid dysfunction |
| LiuJL 2016 | 80 40/40 | S: 2/40  W: 4/40 | NR | NR |
| ZhaoDY 2015 | 120 60/60 | 0 | None for abnormal  of BUS Rt, hepatic and renal function | None for abnormal  of BUS Rt, hepatic and renal function |

**Abbreviations:** S, Shengmai San; W, western medicine; cWM, certain western medicine; BUS, blood, urine and stool; Rt, routine test; NR, not reported.


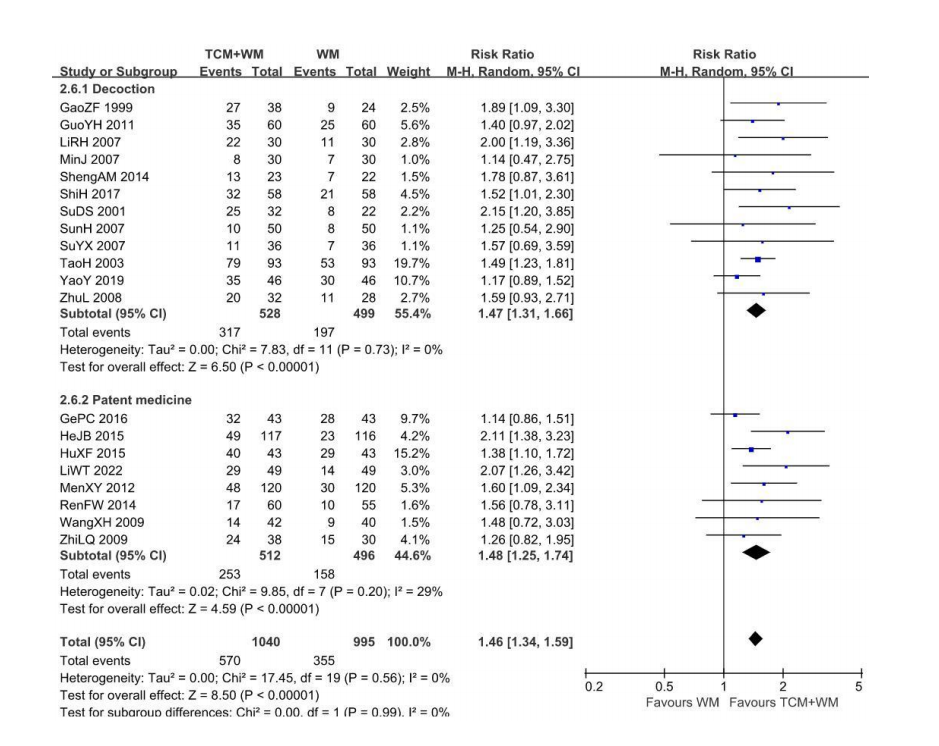


**Supplementary Fig.1** Forest plot of subgroup analysis according to dosage forms for ECG recovery rate in comparison of SMS + WM vs WM


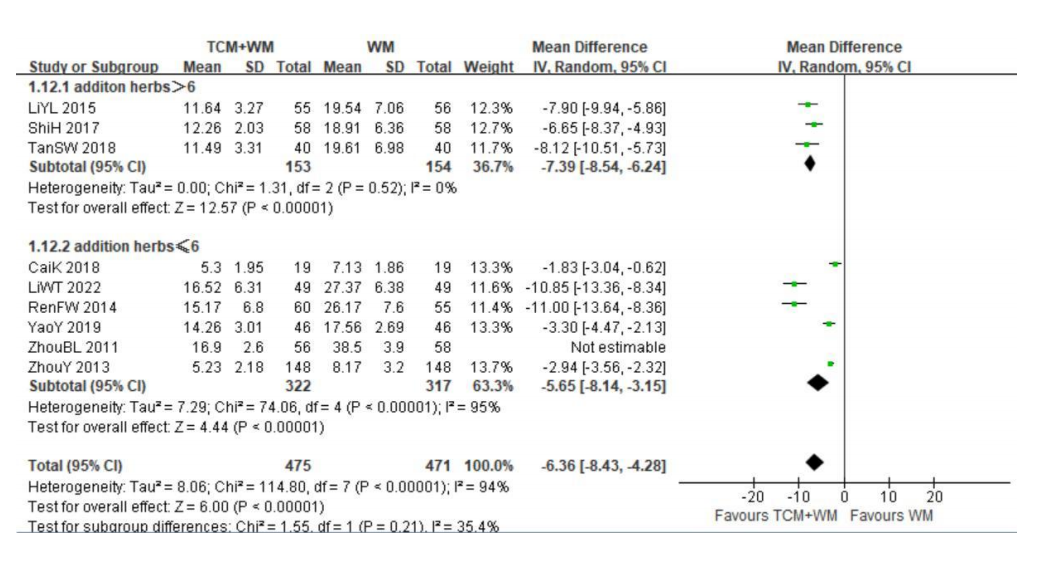


**Supplementary Fig.2** Forest plot of subgroup analysis according to additional herbs’ quantity for CK-MB in comparison of SMS + WM vs WM


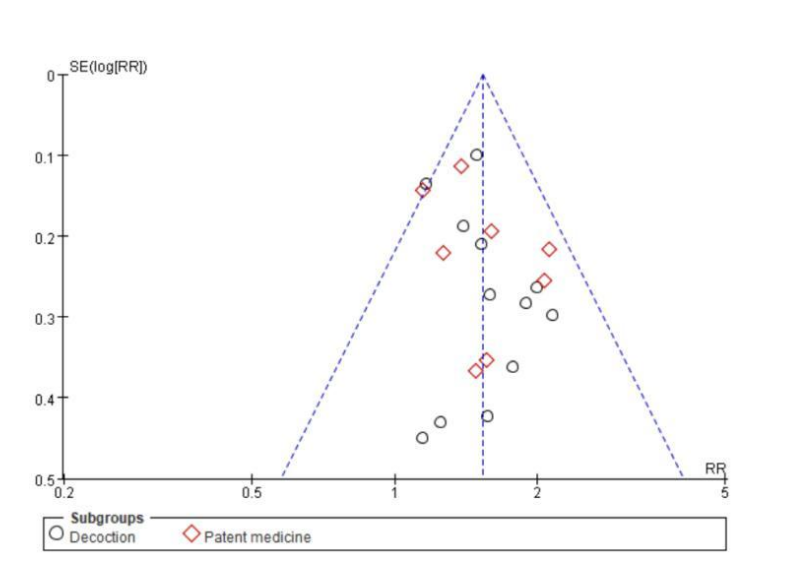


**Supplementary Fig.3** Funnel plot for ECG recovery rate in comparison of SMS + WM vs WM (20 RCTs)


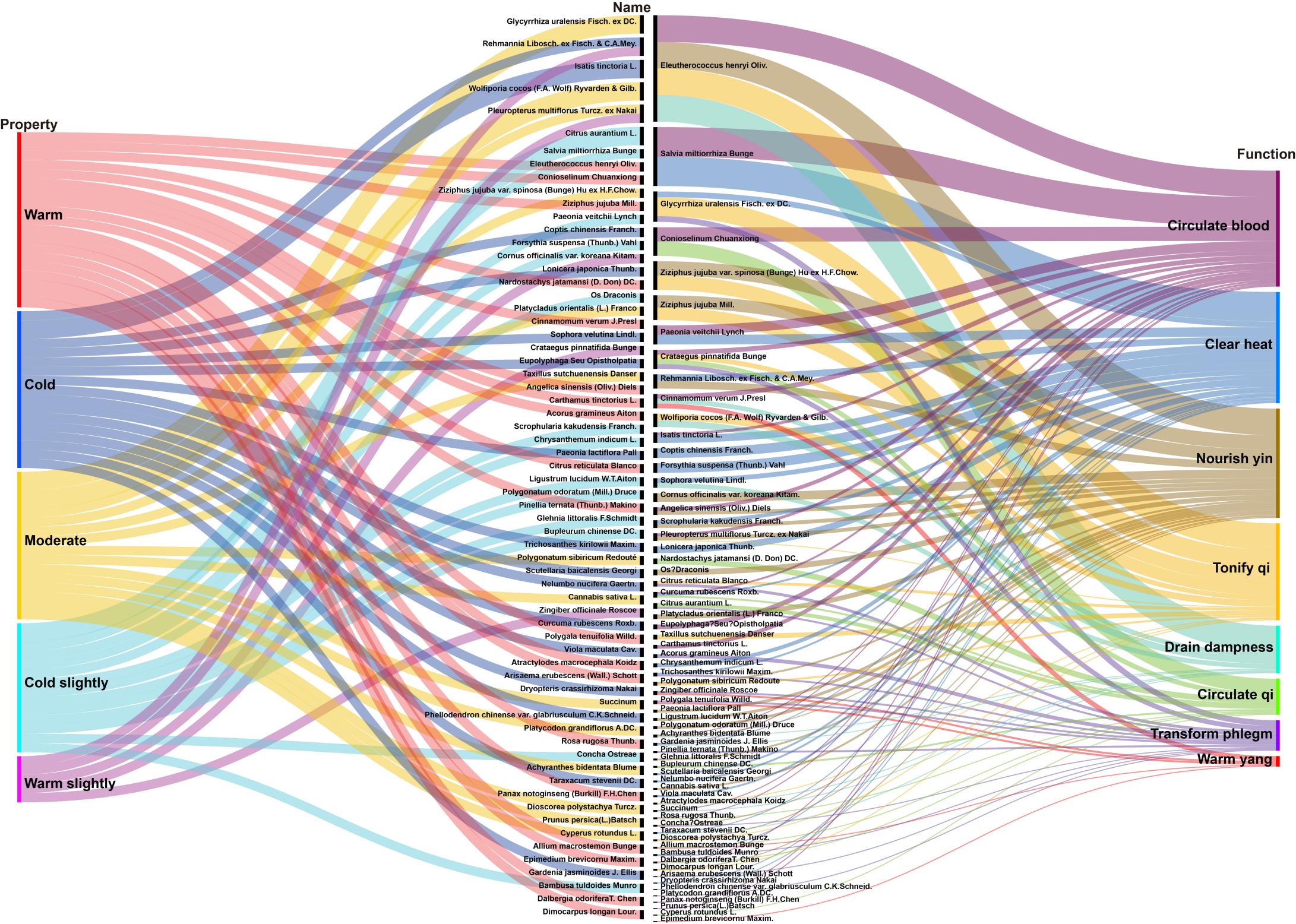


**Supplementary Fig. 4 Names, property and function of auxiliary herbs (the width of band represents the frequency of use)**
